# Supplementary material for: Neural superstatistics for Bayesian estimation of dynamic cognitive models
Source: Sci Rep. 2023 Aug 23;13:13778. doi: 10.1038/s41598-023-40278-3 (PMC10447473; doi:10.1038/s41598-023-40278-3)
Supplement: Supplementary file 1 — Supplementary Information. [file 41598_2023_40278_MOESM1_ESM.pdf]

Appendix

Implementation Details

All experiments, neural networks, and simulation models are implemented using the BayesFlow library `https://github.com/stefanradev93/BayesFlow` built on top of TensorFlow [75]. Code and further instructions for reproducing the results from all experiments and applications in the current manuscript is available at `https://github.com/bayesflow-org/Neural-Superstatistics`.

Stan Benchmark Study

Data Simulation

To simulate the 100 data sets each consisting of $T = 100$ trials, we used the standard DDM implementation (cf. equation (15) in the main text). The diffusion constant was fixed to 1 and the starting point parameter to 0.5 (i.e., symmetric starting point between the two decision boundaries). For data simulation, we randomly sampled parameter sets from the following prior distributions:

$$
\begin{aligned}
v &\sim \Gamma(5.0, \frac{1}{1.3}) \\
a &\sim \Gamma(4.0, \frac{1}{3}) \\
\tau &\sim \Gamma(1.5, \frac{1}{5})
\end{aligned}
$$

where $\Gamma(a, b)$ denotes a Gamma distributions with shape a as the first and scale b as the second argument.

Non-Stationary DDM fitting

We fitted a separate non-stationary DDM with a Gaussian random walk transition model to all 100 simulated data sets. The same implementation and likelihood was used for Stan and our neural estimation method. However, all 3 parameters were allowed to vary according to a Gaussian random walk (cf. equation (3) in the main text). The starting values were sampled from the same prior distributions as in simulation. The hyperparameters of the random walk transition model were sampled from the following distribution:

$$
s_v, s_a, s_\tau \sim \mathcal{B}(1, 25)
$$

where $\mathcal{B}(\alpha, \beta)$ denotes a Beta distribution with α and β parameters. In order to avoid implausible parameter values, the time-varying parameters v_t, a_t, τ_t were clipped to lower bounds $[0, 0, 0]$ and upper bounds $[6, 4, 2]$, respectively.

We trained the neural approximator via online learning (i.e., simulations on the fly) for 50 epochs with 1000 iterations each and a batch size of 8. We use an Adam optimizer with an initial learning rate of 5×10^{-4} and a cosine learning rate decay schedule. After training the network, we draw 4000 posterior samples (the same as with Stan) for each of the 100 data sets.

Simulation Study

In what follows, we describe the settings for the four different simulation scenarios, namely, the static DDM, the DDM with stationary variability, the DDM with non-stationary variability, and the static DDM with random uniform jumps at pre-defined time steps (i.e., regime switching DDM). For each scenario, we simulated 200 data sets, each consisting of $T = 400$ time steps.

We trained the neural approximator via online learning (i.e., simulations on the fly) for 75 epochs with 1000 iterations each and a batch size of 8. We use an Adam optimizer with an initial learning rate of 5×10^{-4} and a cosine learning rate decay schedule. After training the network, we draw 4000 posterior samples (the same as with Stan) for each of the 100 data sets.

Static DDM

To simulate the 200 data sets for the static DDM scenario, we used the same prior and likelihood as in the **Stan Benchmark Study**.

Stationary Variability DDM

For the stationary variability DDM, we used the same DDM implementation as in the static DDM scenario except that we used the following variability statements:

$$
\begin{aligned}
v_t &\sim \mathcal{N}(v, v_s) \\
a_t &\sim \mathcal{N}(a, a_s) \\
\tau_s &\sim \mathcal{U}(\tau - \frac{\tau_s}{2}, \tau + \frac{\tau_s}{2})
\end{aligned}
$$

where $\mathcal{N}(\mu, \sigma)$ denotes a Normal distribution with location μ and standard deviation σ and $\mathcal{U}(\text{lower, upper})$ denotes an Uniform distribution with a lower and a upper bound.

The newly introduced variability parameters (v_s, a_s, τ_s) were sampled from the following prior distributions:

$$
v_s, a_s, \tau_s \sim \mathcal{TN}_{[0,\text{inf}]}(0, 0.1)
$$

where $\mathcal{TN}_{[a,b]}(\mu, \sigma)$ denotes the truncated normal distribution with location μ and standard deviation σ truncated within the interval $[a, b]$.

To avoid implausible values the per trial parameters v_t, a_t, τ_t were bounded with lower bounds [0, 0, 0] and upper bounds [6, 4, 2] respectively.

Non-Stationary DDM

We used the same non-stationary DDM implementation as described in **Stan Benchmark Study**.

Regime Switching DDM

The regime switching DDM is basically the same implementation as the static DDM, but the parameter jumped uniformly at 3 specific time steps ($T = 100; T = 200; T = 300$) and stayed again constant after the jump:

$$\theta_t = \begin{cases} \theta_{t-1}, & \text{if } t \notin 100, 200, 300 \\ \mathcal{U}(\text{lower, upper}), & \text{if } t \in \{100, 200, 300\} \end{cases} \tag{17}$$

where the lower and upper bounds of the Uniform distributions are [0, 0, 0] and [6, 4, 2], respectively. The starting values of the parameters were once again sampled form the same prior distributions as in the static DDM.

Amortized inference

We fitted the same non-stationary DDM with a Gaussian transition model as described above to all four scenarios. To train the networks we used 75 epochs with 1000 iterations each and a batch size of 16. After training the network we fitted the model to each simulation of each scenario separately and obtained 2000 posterior samples.

True vs. Estimated Parameters

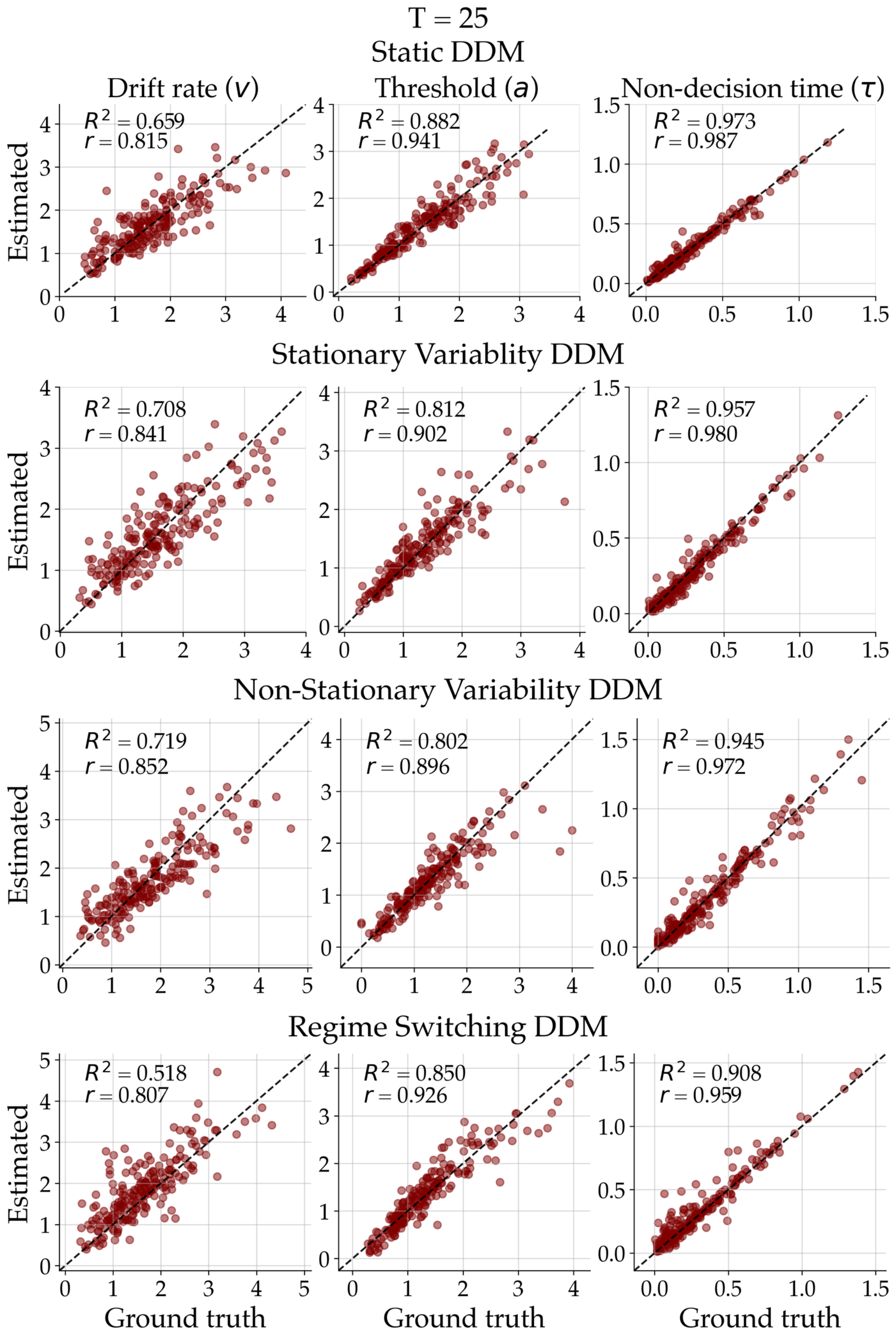

Figure A.10: True data generating parameters plotted against posterior means for all 3 parameters and simulation scenarios separately at time point $T = 25$.

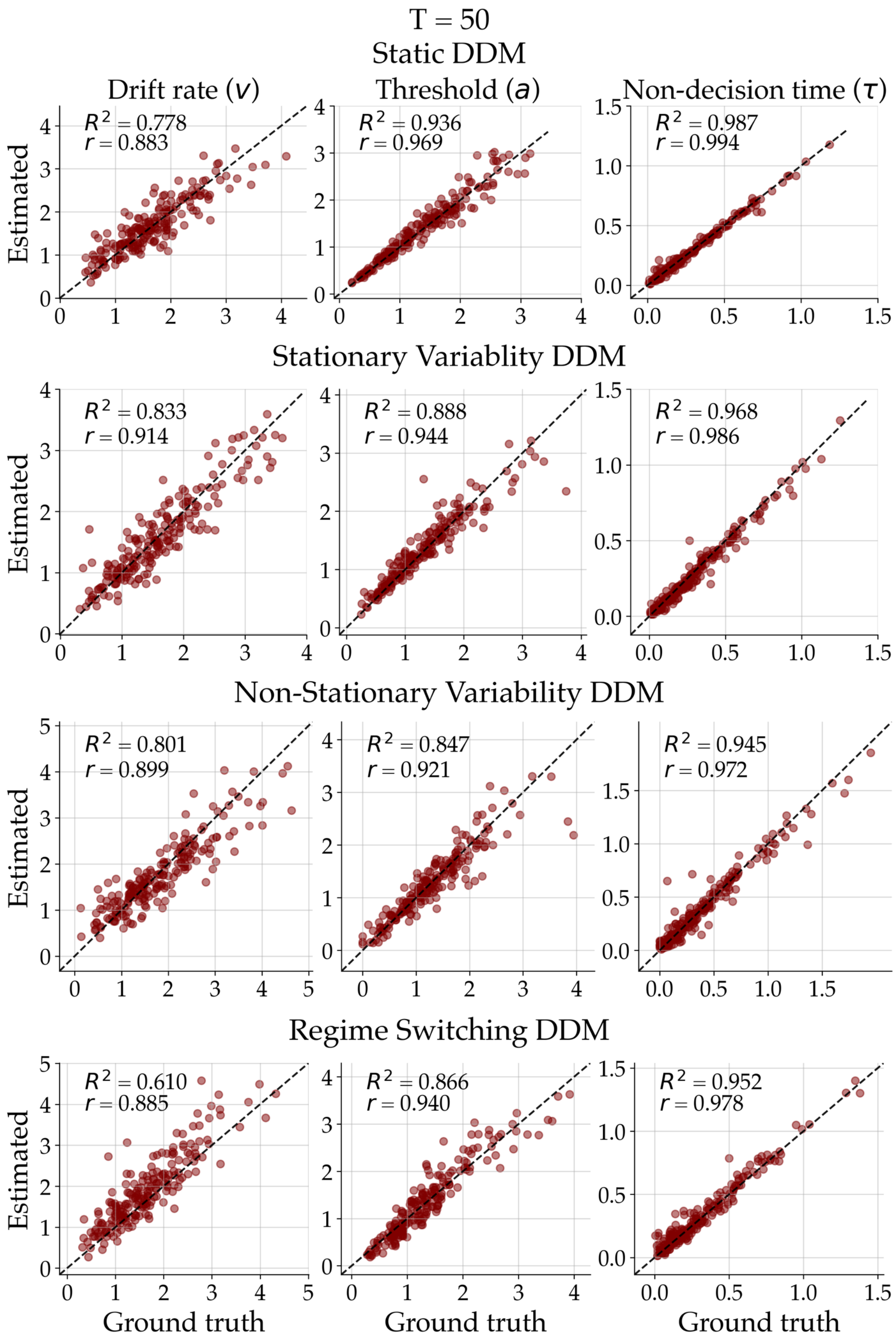

Figure A.11: True data generating parameters plotted against posterior means for all 3 parameters and simulation scenarios separately at time point $T = 50$.

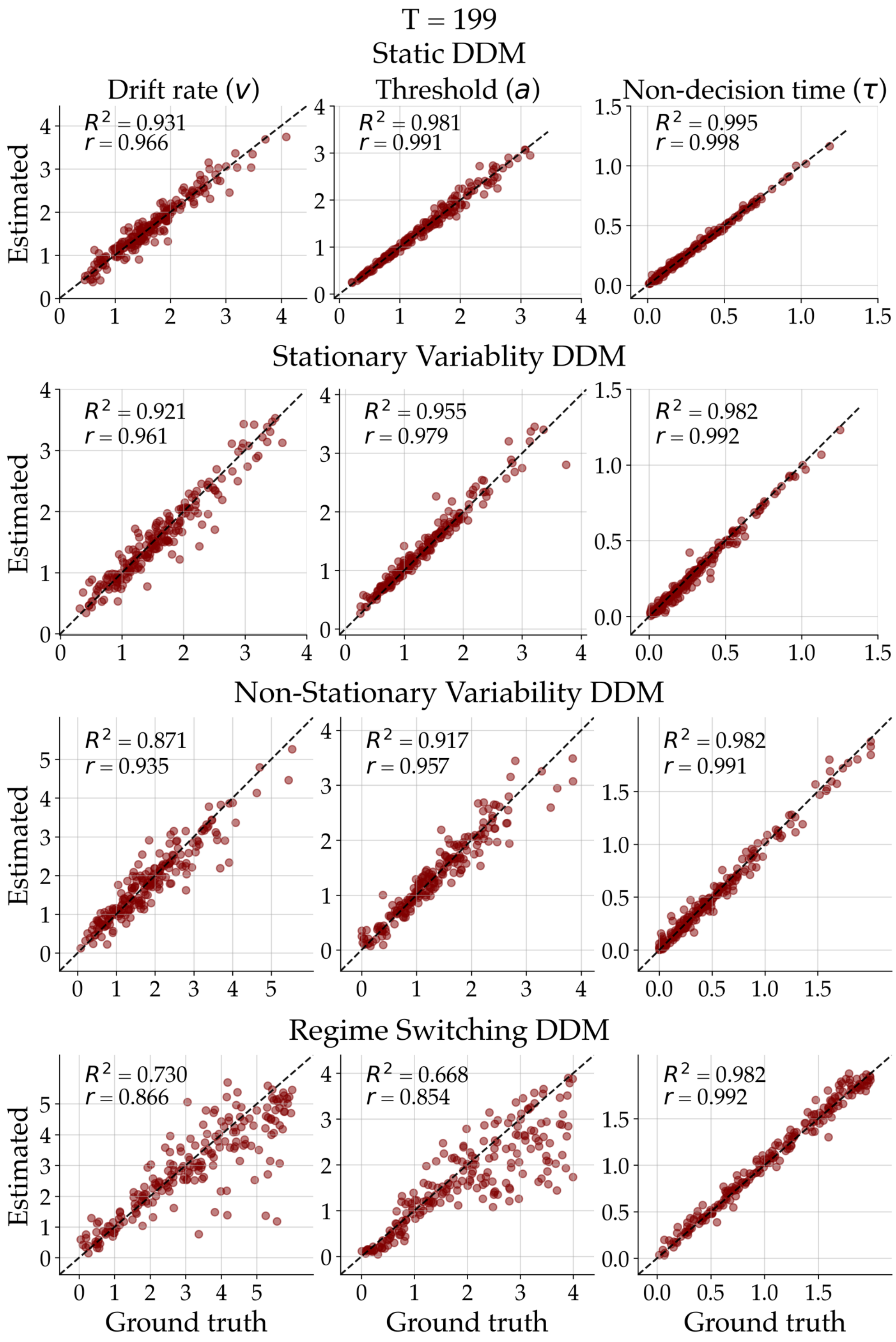

Figure A.12: True data generating parameters plotted against posterior means for all 3 parameters and simulation scenarios separately at time point $T = 199$.

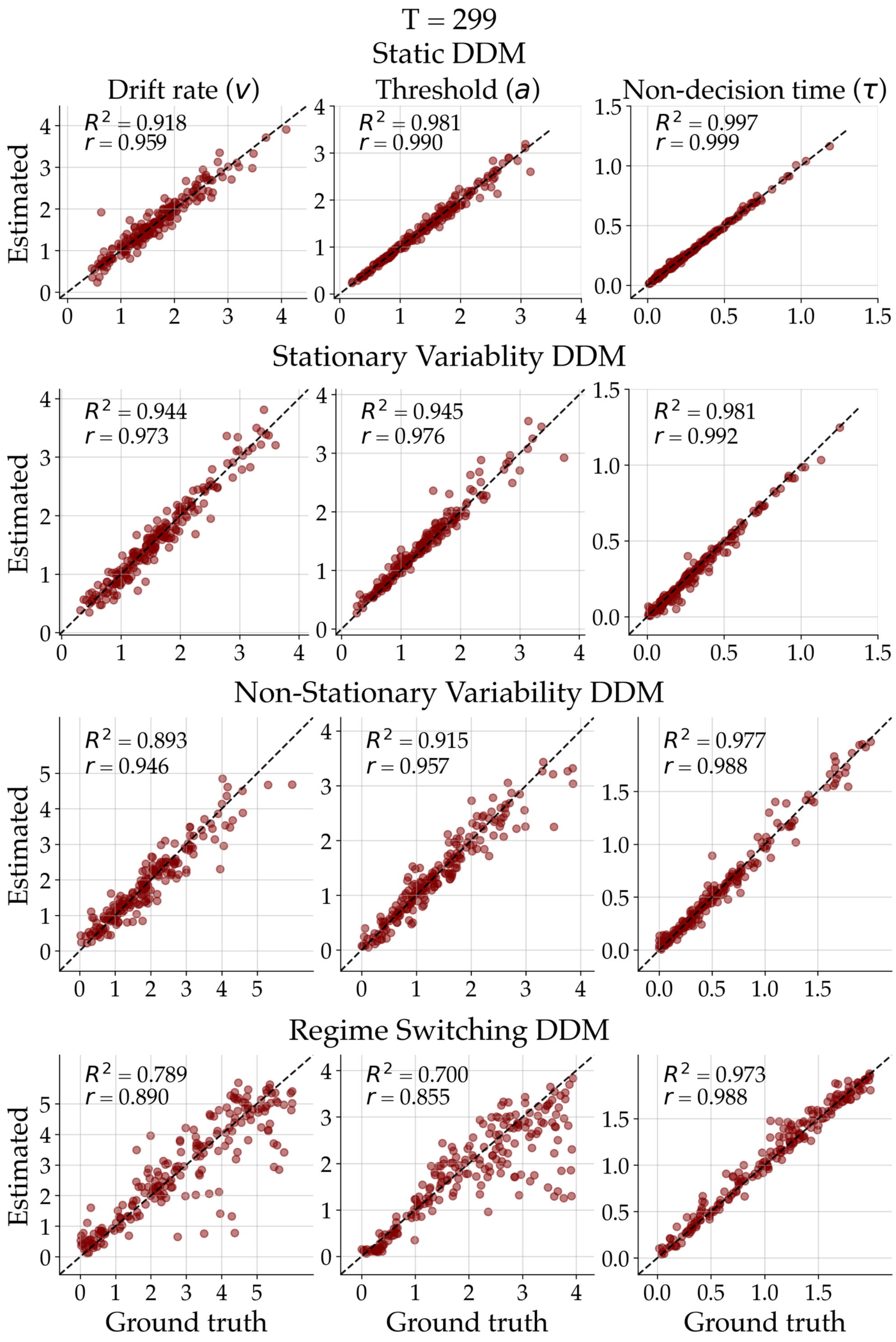

Figure A.13: True data generating parameters plotted against posterior means for all 3 parameters and simulation scenarios separately at time point $T = 299$.

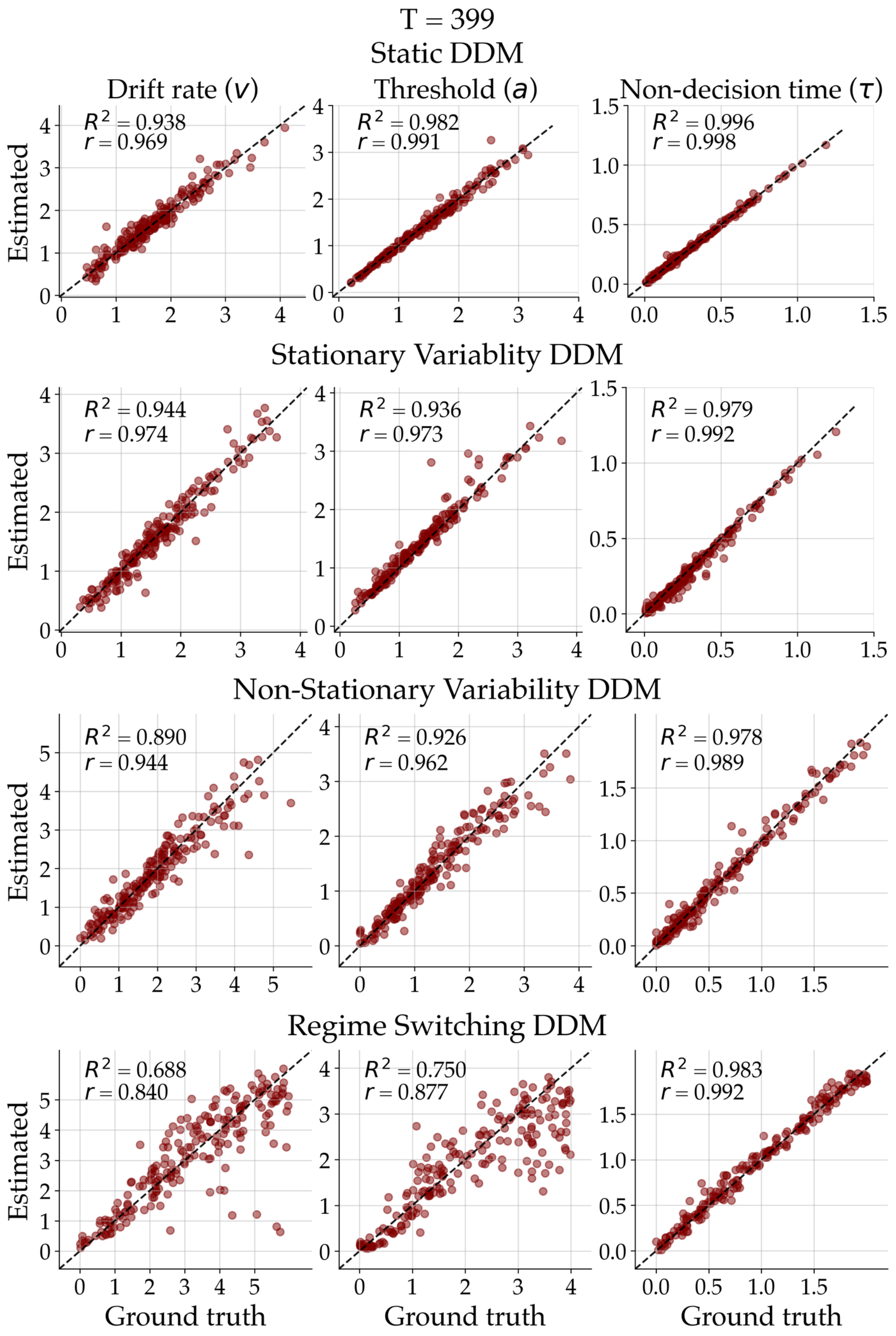

Figure A.14: True data generating parameters plotted against posterior means for all 3 parameters and simulation scenarios separately at time point $T = 399$.

Mean Absolute Error

As an additional analysis of the overall parameter recovery performance of the non-stationary DDM, we computed the median absolute error (MAE) between the true data generating parameter and the posterior mean for all DDM parameters and simulation scenarios separately. In the top row of Figure A.15 we can see that the posterior estimates of the non-stationary DDM quickly approach the true data-generating parameter when the true parameter was constant over time. That said, there remains some error between the true and estimated parameter even after 400 time steps. This error is the largest in the drift rate parameter (≈ 0.15). We see similar recovery performance in the scenario, where the parameters were allowed to randomly fluctuate around a constant value (second row in Figure A.15). However, we observe a larger variability in the MAE. The third row depicts the MAE when data was simulated with the same model as we fitted to the data (i.e., the well-specified case). Once again, the MAE quickly decreases in the beginning and then flattens out. However, in this scenario, the MAE remains on a larger level than in the previous two scenarios. Also, the variability of the MAE between data fits is larger. This is not surprising because the estimation of non-stationary model parameters is more difficult than static or stationary variable parameters. The last row in Figure A.15 shows how the parameter estimates of the non-stationary DDM react to sudden jumps in otherwise constant parameters. We observe that the MAE significantly increases when a jump occurred and then decreases again.

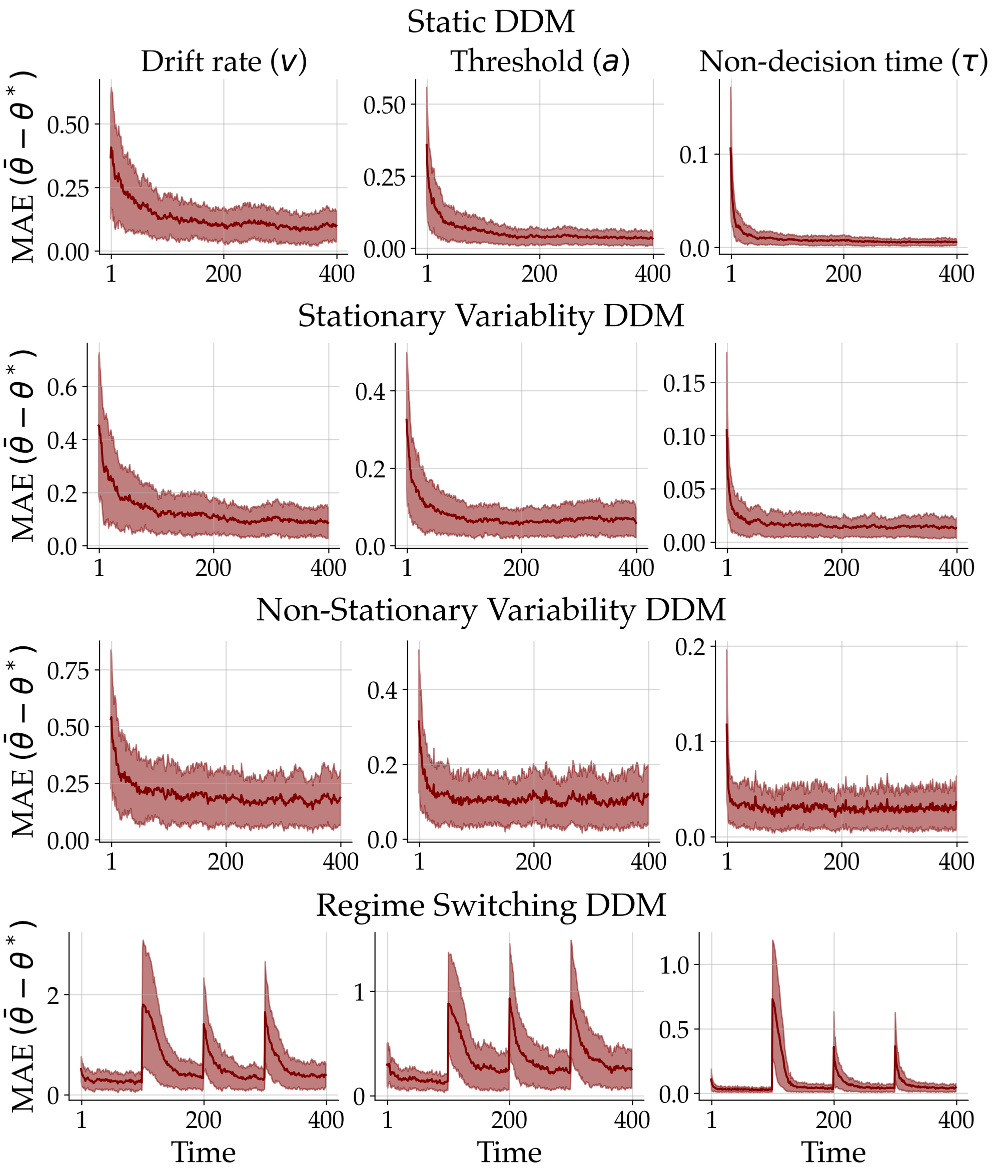

Figure A.15: Median absolute error (MAE) between the data generating parameters and the estimated posterior means aggregated across the 200 simulations over time for each DDM parameter (columns) and simulation scenario (rows) separately. The red shaded areas depict the median absolute deviation of the absolute errors.

Human Data Application: Random-Dot Motion

We fitted a non-stationary DDM with a Gaussian random walk transition model to each individual in the data set separately. The model implementation was the same as in the **Stan Benchmark** and the **Simulation Study**. For the training of our neural estimation method we used 75 epoch each consisting of 1000 iteration with a batch size of 16. After training we obtained 2000 posterior samples.

Human Data Application: Lexical Decision

Gaussian Process DDM

For the Gaussian Process transition model, we first create a T x T squared distance matrix with $T = 3200$. Based on this distance matrix we calculate the radial basis function kernel (cf. equation (6) in the main text) given the two parameters, amplitude σ and length-scale l, resulting in the covariance k for the multivariate normal distribution of the Gaussian Process:

$$\theta_{1:T} \sim \mathcal{MVN}(\mu_\theta, k)$$

where μ_θ is the mean parameter value. For these means we used the same priors we otherwise used for the starting values of the DDM parameters ($v_{0,i}$, a_0, τ_0). In the following we present a list of the priors used by the Gaussian Process DDM simulator to generate data for the simulation study and for training the neural networks. $\Gamma(a, b)$ refers to a Gamma distribution parameterized with shape a and scale b. The same prior distribution was used for all $i = 4$ drift rates $v_{0,i}$. $\mathcal{U}(a, b)$ stands for a continuous uniform distribution with a lower limit a and an upper limit b. l_j denotes the length-scale parameters of the GP transition model. The same prior distribution was used for all $j = 1, \ldots, 6$ length-scale parameters governing the transitions of the DDM parameters. The amplitude parameter σ of the Gaussian kernel is usually highly correlates with the length-scale l. Thus, we fixed σ to sensible values for all low-level parameter transitions.

$$\begin{aligned}
v_{0,i} &\sim \Gamma(2.5, \frac{1}{1.5}) \\
a_0 &\sim \Gamma(4.0, \frac{1}{3}) \\
\tau_0 &\sim \Gamma(1.5, \frac{1}{5}) \\
l_j &\sim \mathcal{U}(0.1, 10) \\
\sigma_{v_{1:4}} &= 0.15 \\
\sigma_a &= 0.1 \\
\sigma_\tau &= 0.05
\end{aligned}$$

Simulation-Based Calibration

We validate the computational faithfulness of our Bayesian inference algorithm using simulation-based calibration, a robust method for ensuring unbiased posterior distributions. The underlying principle is that an ensemble of posterior distributions should be indistinguishable from the prior distribution. To accomplish this, we carry out 2000 simulations with the dynamic DDM, each generating a separate data set. For each simulated data set, we fit the model and obtain 250 posterior samples. These posterior distributions collectively form an ensemble.

When we calculate rank statistics for the ensemble relative to the prior distribution then these should be uniformly distributed. To assess the uniformity at predefined time points, we utilize the empirical cumulative distribution function (ECDF) for each marginal rank distribution. Comparing it with a uniform ECDF allows us to gauge how the data is distributed. We further draw ECDF simultaneous bands using simulations from the uniform, providing an intuitive graphical test for uniformity. For clarity, Figure A.16 presents the ECDF difference, providing a more dynamic range for the visualization. The red line (ECDF difference) should consistently fall within the gray shaded area (confidence band) across the entire range of fractional rank statistic values. In the majority of cases, this criterion is met for most parameters at all selected time points. Some slight deviations are observed for the threshold and non-decision parameters; however, these are typically small and not a major cause for concern.

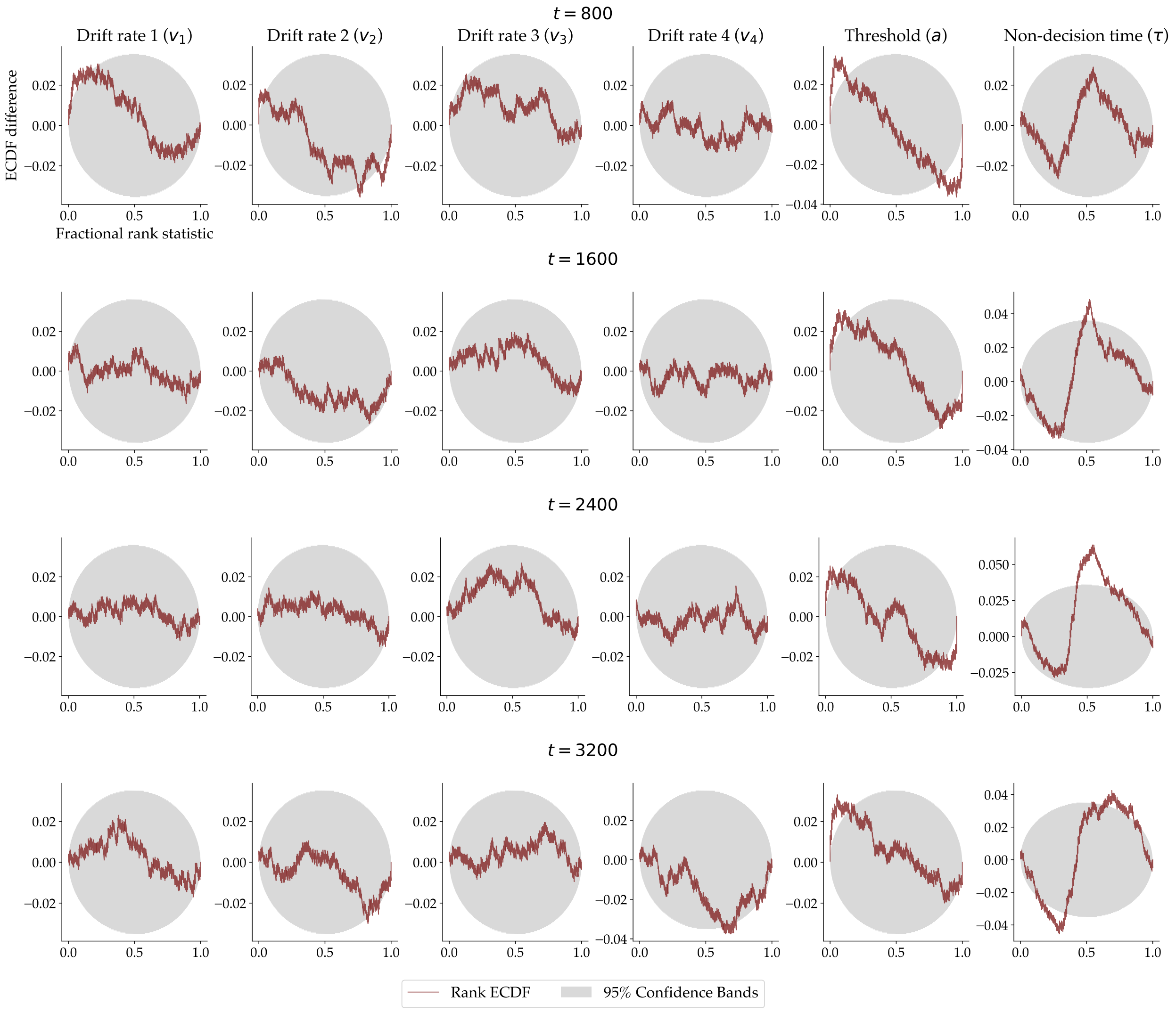

Figure A.16: **ECDF difference plot** 95% simultaneous confidence bands (gray) for the empirical cumulative distribution function (ECDF; red) for all 6 parameters at four selected time points (800, 1600, 2500, 3200) separately.

Parameter Recovery Study

A simulation study was performed to probe the dynamic DDM's capability of recovering data-generating parameter dynamics. To this end, we simulated 1000 data sets with the dynamic DDM and fit it to these data. The following figures show posterior predictions of 3 randomly selected simulated data sets and the comparison between the inferred and the true data-generating low-level parameter dynamics. The parameter recovery performance across all 1000 data sets over all 3200 time points for all 6 model parameters can be inspected as a GIF in our GitHub repository (https://github.com/bayesflow-org/Neural-Superstatistics)

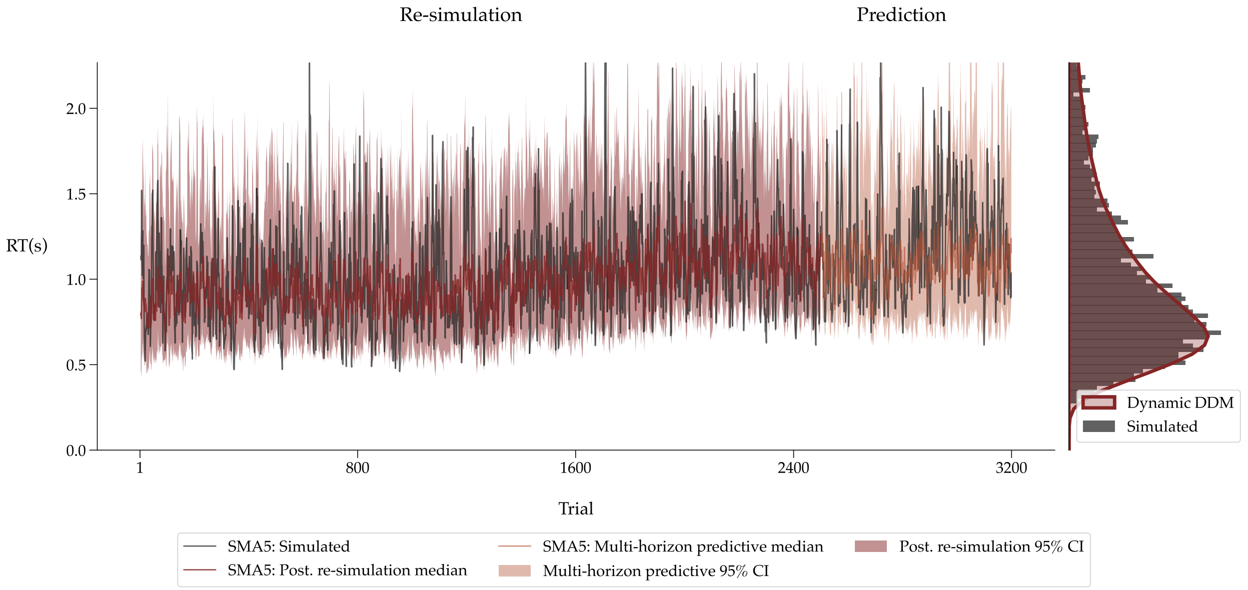

Figure A.17: **Left panel** The simulated RT time series is shown in black. From trial 1 to 2500, the median posterior re-simulation (aka *retrodictive check*) using the dynamic DDM is shown in red. The models' multi-horizon prediction is depicted for the remaining trials in orange. The shaded areas for the posterior re-simulation and prediction correspond to the 95% credibility interval. All the time series were smoothed via a simple moving average (SMA) with a period of 5. **Right panel** The raw simulated RT distribution is plotted as a histogram in black. The re-simulated RT distributions from the dynamic DDM are shown as kernel density estimates (KDEs) in red.

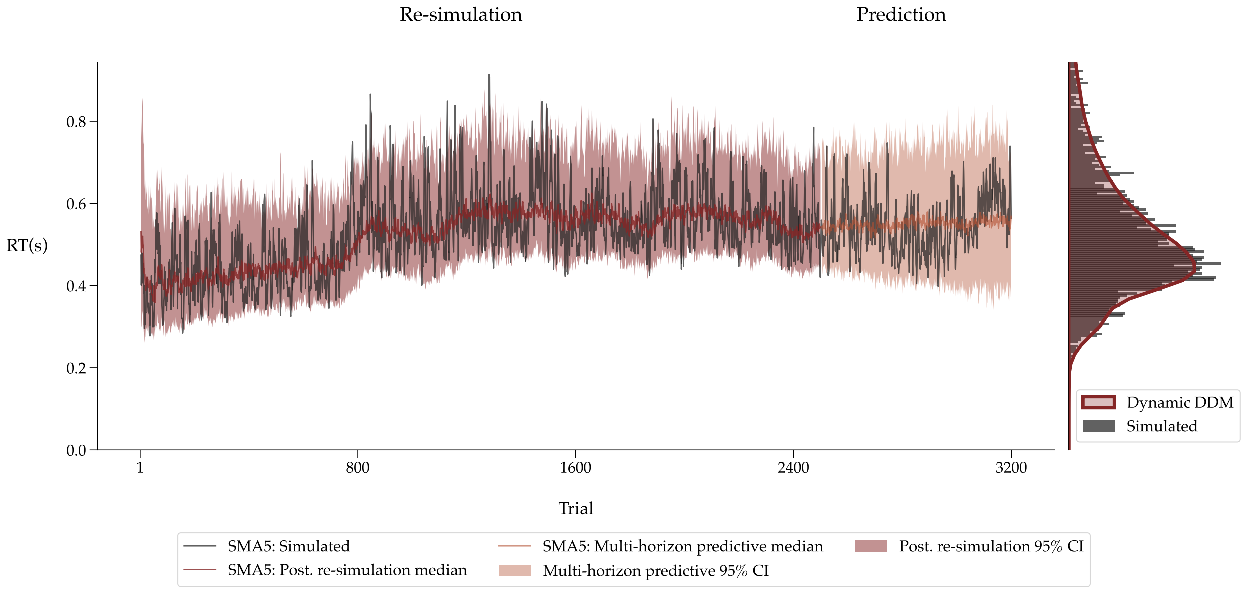

Figure A.18: **Left panel** The simulated RT time series is shown in black. From trial 1 to 2500, the median posterior re-simulation (aka *retrodictive check*) using the dynamic DDM is shown in red. The models' multi-horizon prediction is depicted for the remaining trials in orange. The shaded areas for the posterior re-simulation and prediction correspond to the 95% credibility interval. All the time series were smoothed via a simple moving average (SMA) with a period of 5. **Right panel** The raw simulated RT distribution is plotted as a histogram in black. The re-simulated RT distributions from the dynamic DDM are shown as kernel density estimates (KDEs) in red.

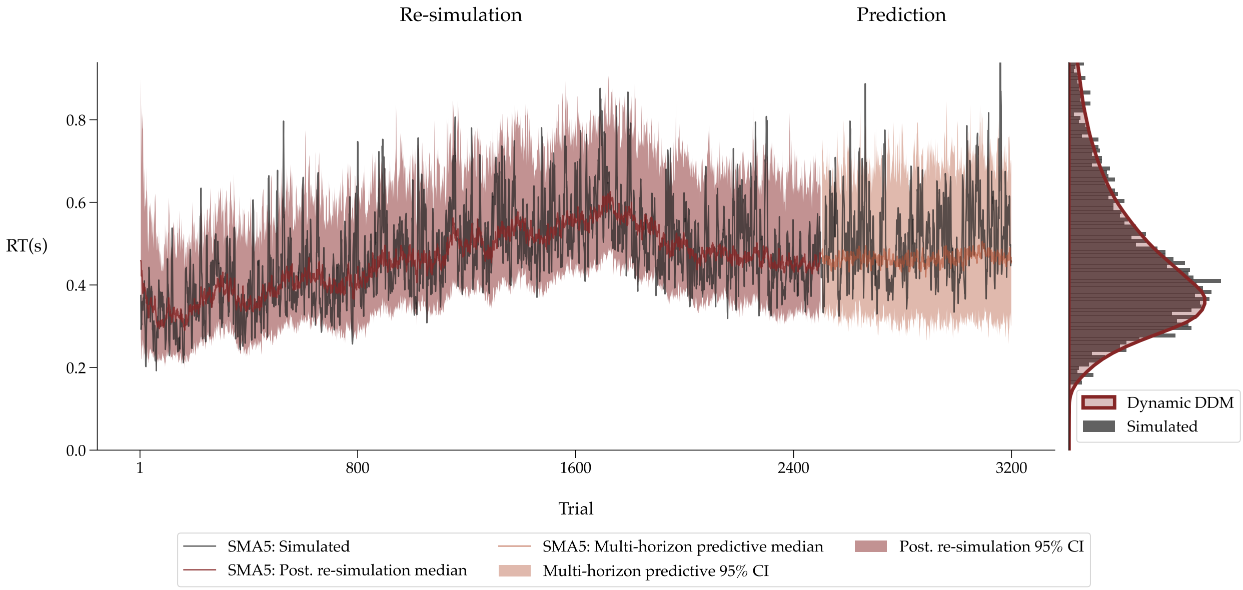

Figure A.19: **Left panel** The simulated RT time series is shown in black. From trial 1 to 2500, the median posterior re-simulation (aka *retrodictive check*) using the dynamic DDM is shown in red. The models' multi-horizon prediction is depicted for the remaining trials in orange. The shaded areas for the posterior re-simulation and prediction correspond to the 95% credibility interval. All the time series were smoothed via a simple moving average (SMA) with a period of 5. **Right panel** The raw simulated RT distribution is plotted as a histogram in black. The re-simulated RT distributions from the dynamic DDM are shown as kernel density estimates (KDEs) in red.

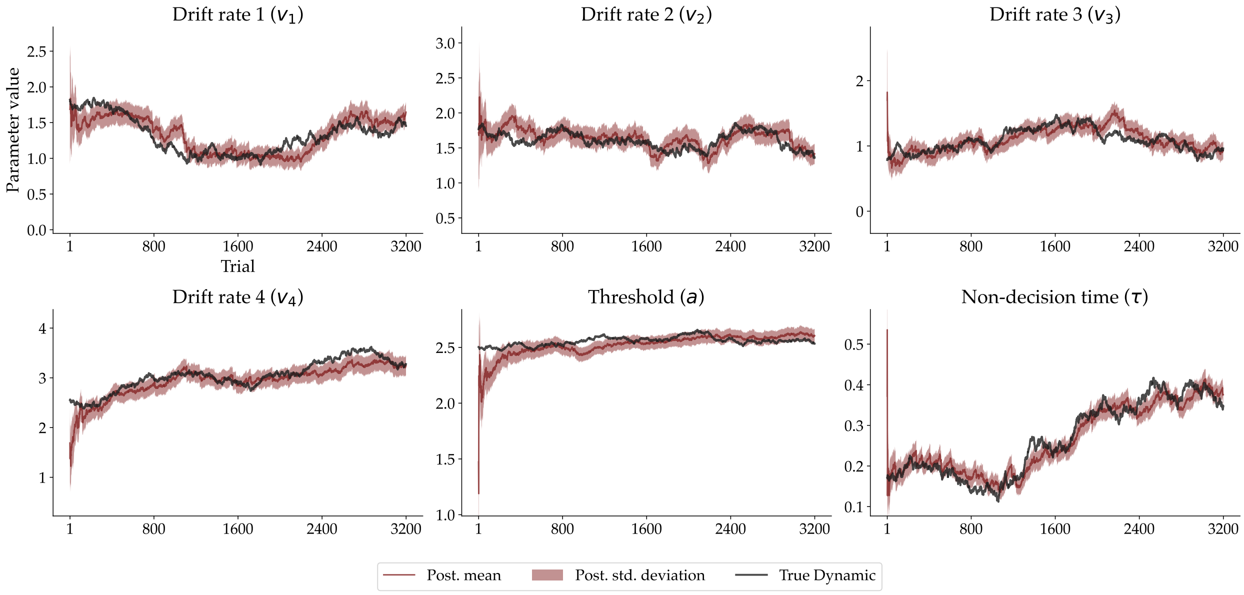

Figure A.20: The trial-wise posterior mean and ± 1 standard deviation for all six parameters, namely the four drift rates v_1 - v_4 (one for each experimental condition), the threshold a, and the non-decision time τ in red. The true data generating parameter dynamic in black.

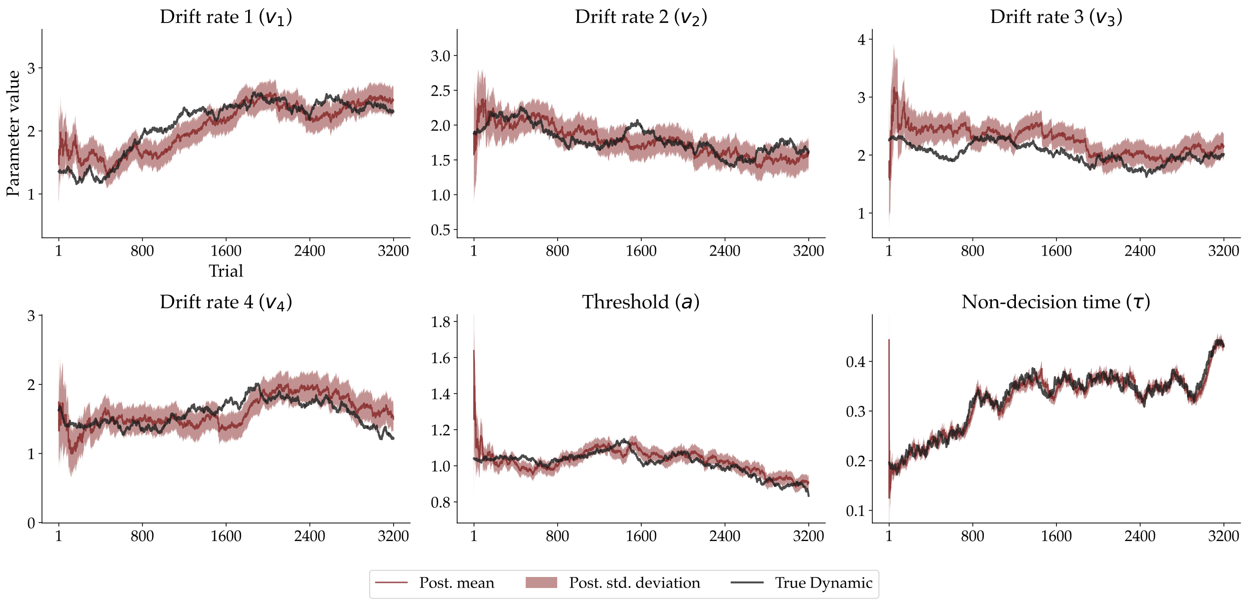

Figure A.21: The trial-wise posterior mean and ± 1 standard deviation for all six parameters, namely the four drift rates v_1 - v_4 (one for each experimental condition), the threshold a, and the non-decision time τ in red. The true data generating parameter dynamic in black.

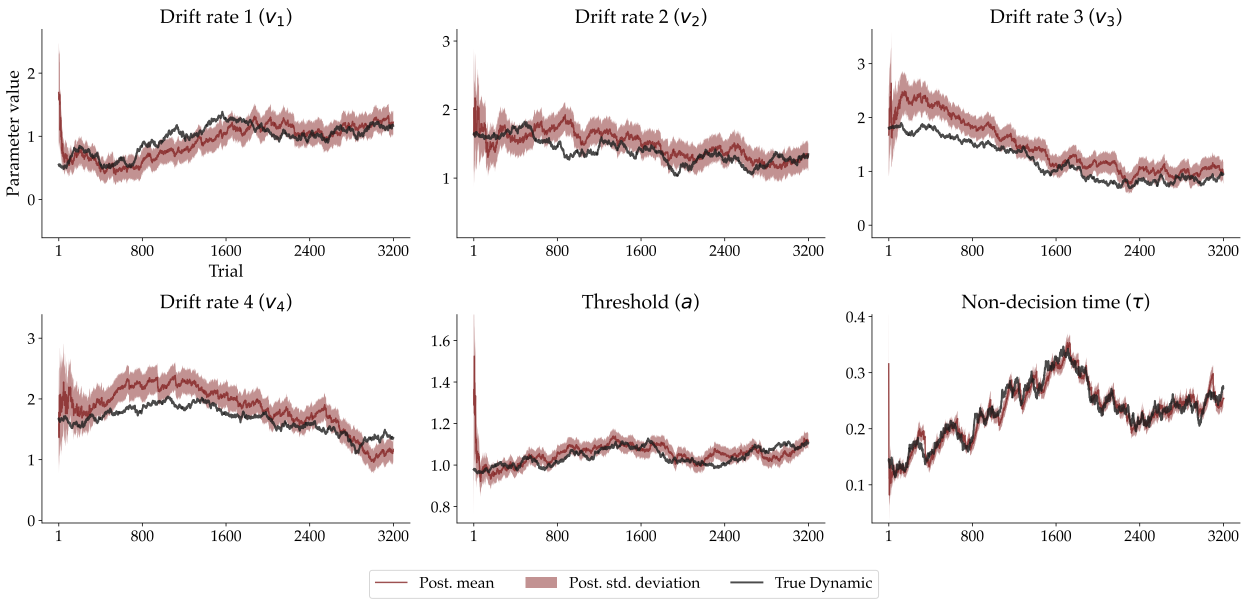

Figure A.22: The trial-wise posterior mean and ± 1 standard deviation for all six parameters, namely the four drift rates v_1 - v_4 (one for each experimental condition), the threshold a, and the non-decision time τ in red. The true data generating parameter dynamic in black.

Individual Model Fits and Predictions

In the following, we show the fit and multi-horizon predictions of the dynamic DDM on the individual data of the remaining 10 participants not shown in the main text.

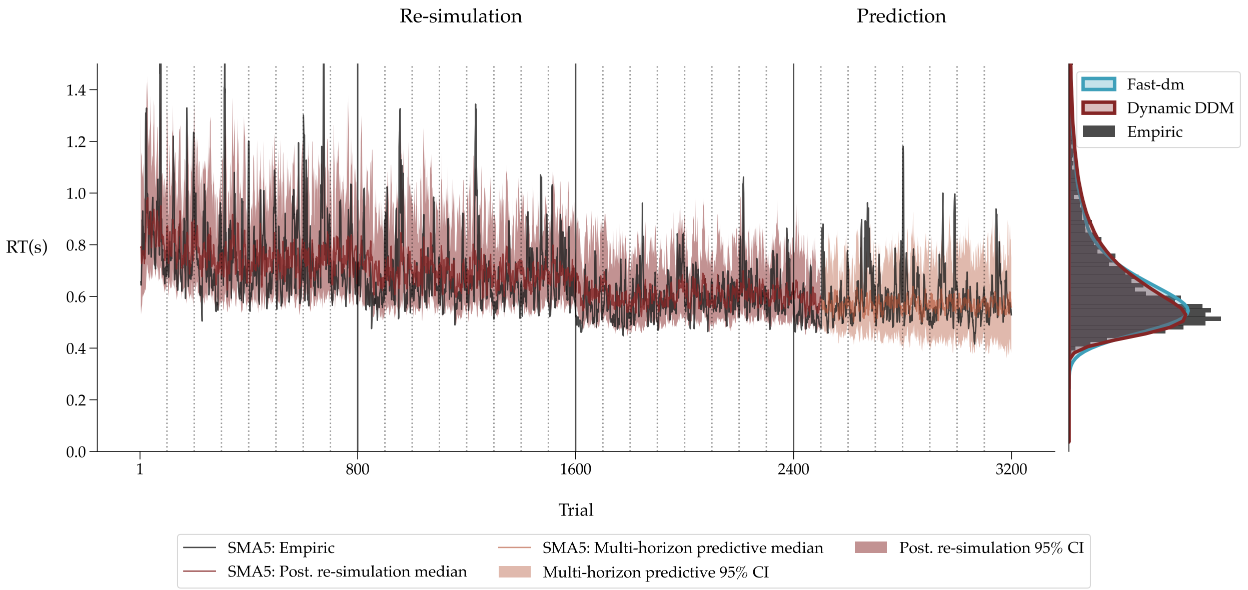

Figure A.23: **Left panel** The empirical RT time series of a single individual in black. From trial 1 to 2500, the median posterior re-simulation (aka *retrodictive check*) using the dynamic DDM is shown in red. The models' multi-horizon prediction is depicted for the remaining trials in orange. The shaded areas for the posterior re-simulation and prediction correspond to the 95% credibility interval. All the time series were smoothed via a simple moving average (SMA) with a period of 5. The dotted vertical lines indicate the end of an experimental block, and the solid vertical lines the end of an experimental session. **Right panel** The raw RT distribution is plotted as a histogram in black. The re-simulated RT distributions from the dynamic DDM and reference re-simulations from the static DDM using `fast-dm` are shown as kernel density estimates (KDEs) in red and blue, respectively.

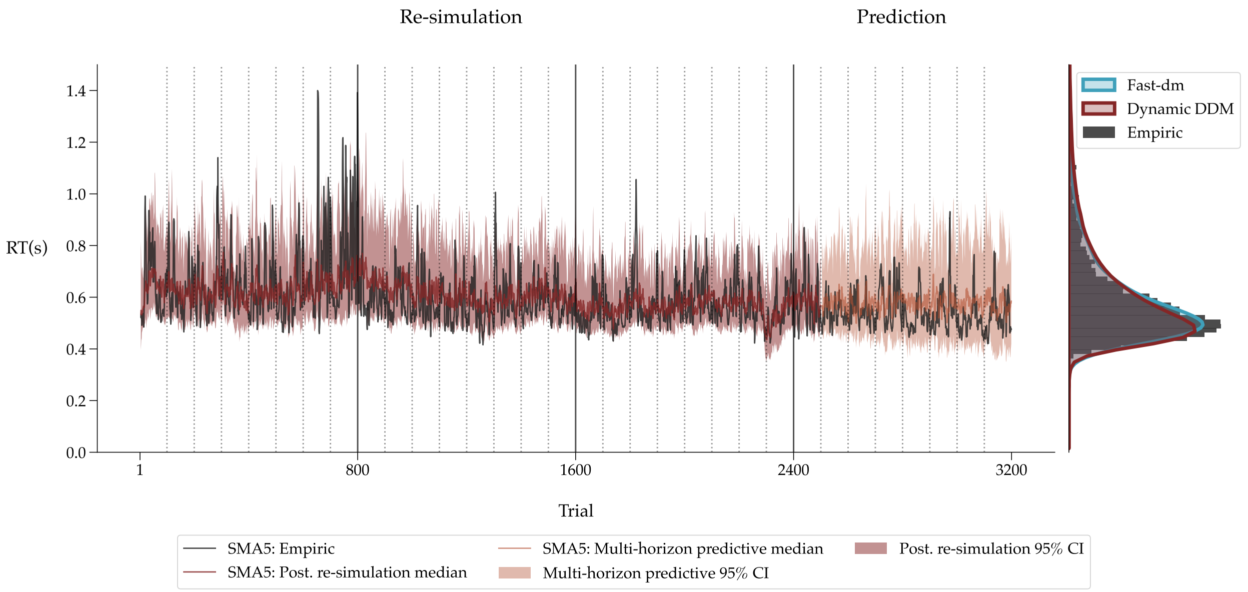

Figure A.24: **Left panel** The empirical RT time series of a single individual in black. From trial 1 to 2500, the median posterior re-simulation (aka *retrodictive check*) using the dynamic DDM is shown in red. The models' multi-horizon prediction is depicted for the remaining trials in orange. The shaded areas for the posterior re-simulation and prediction correspond to the 95% credibility interval. All the time series were smoothed via a simple moving average (SMA) with a period of 5. The dotted vertical lines indicate the end of an experimental block, and the solid vertical lines the end of an experimental session. **Right panel** The raw RT distribution is plotted as a histogram in black. The re-simulated RT distributions from the dynamic DDM and reference re-simulations from the static DDM using `fast-dm` are shown as kernel density estimates (KDEs) in red and blue, respectively.

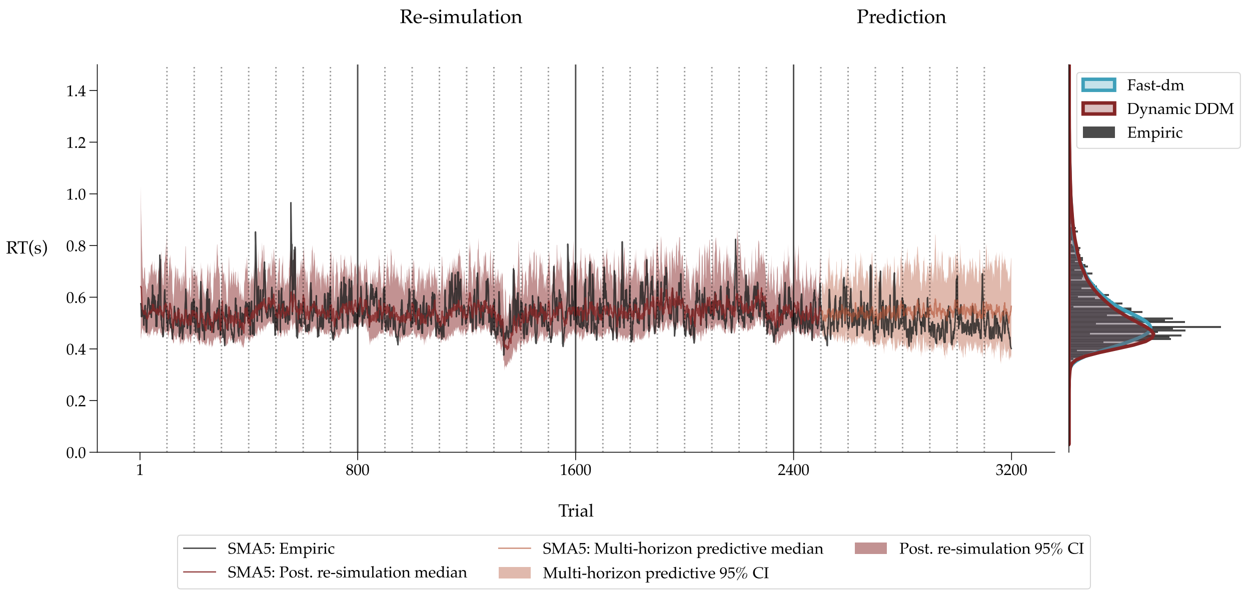

Figure A.25: **Left panel** The empirical RT time series of a single individual in black. From trial 1 to 2500, the median posterior re-simulation (aka *retrodictive check*) using the dynamic DDM is shown in red. The models' multi-horizon prediction is depicted for the remaining trials in orange. The shaded areas for the posterior re-simulation and prediction correspond to the 95% credibility interval. All the time series were smoothed via a simple moving average (SMA) with a period of 5. The dotted vertical lines indicate the end of an experimental block, and the solid vertical lines the end of an experimental session. **Right panel** The raw RT distribution is plotted as a histogram in black. The re-simulated RT distributions from the dynamic DDM and reference re-simulations from the static DDM using `fast-dm` are shown as kernel density estimates (KDEs) in red and blue, respectively.

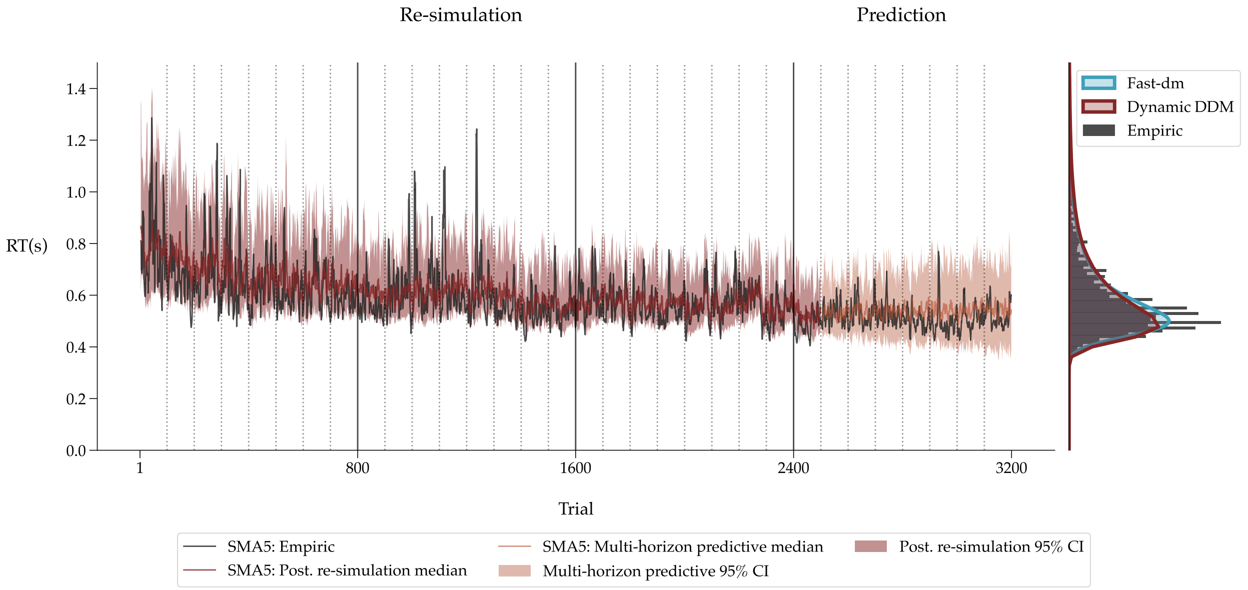

Figure A.26: **Left panel** The empirical RT time series of a single individual in black. From trial 1 to 2500, the median posterior re-simulation (aka *retrodictive check*) using the dynamic DDM is shown in red. The models' multi-horizon prediction is depicted for the remaining trials in orange. The shaded areas for the posterior re-simulation and prediction correspond to the 95% credibility interval. All the time series were smoothed via a simple moving average (SMA) with a period of 5. The dotted vertical lines indicate the end of an experimental block, and the solid vertical lines the end of an experimental session. **Right panel** The raw RT distribution is plotted as a histogram in black. The re-simulated RT distributions from the dynamic DDM and reference re-simulations from the static DDM using `fast-dm` are shown as kernel density estimates (KDEs) in red and blue, respectively.

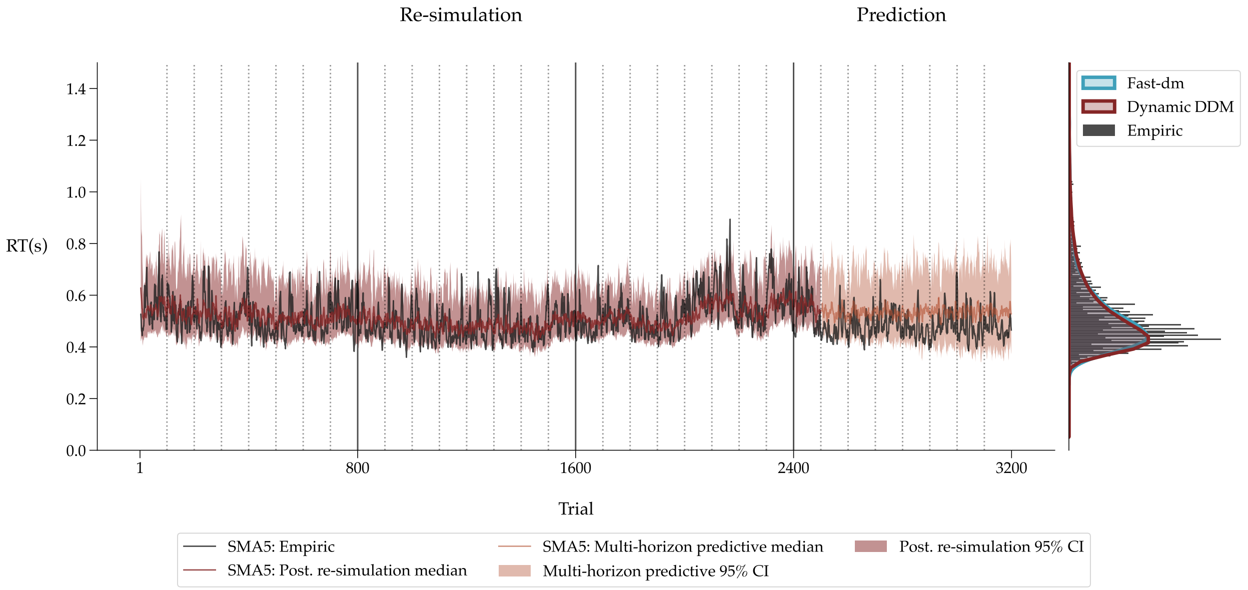

Figure A.27: **Left panel** The empirical RT time series of a single individual in black. From trial 1 to 2500, the median posterior re-simulation (aka *retrodictive check*) using the dynamic DDM is shown in red. The models' multi-horizon prediction is depicted for the remaining trials in orange. The shaded areas for the posterior re-simulation and prediction correspond to the 95% credibility interval. All the time series were smoothed via a simple moving average (SMA) with a period of 5. The dotted vertical lines indicate the end of an experimental block, and the solid vertical lines the end of an experimental session. **Right panel** The raw RT distribution is plotted as a histogram in black. The re-simulated RT distributions from the dynamic DDM and reference re-simulations from the static DDM using `fast-dm` are shown as kernel density estimates (KDEs) in red and blue, respectively.

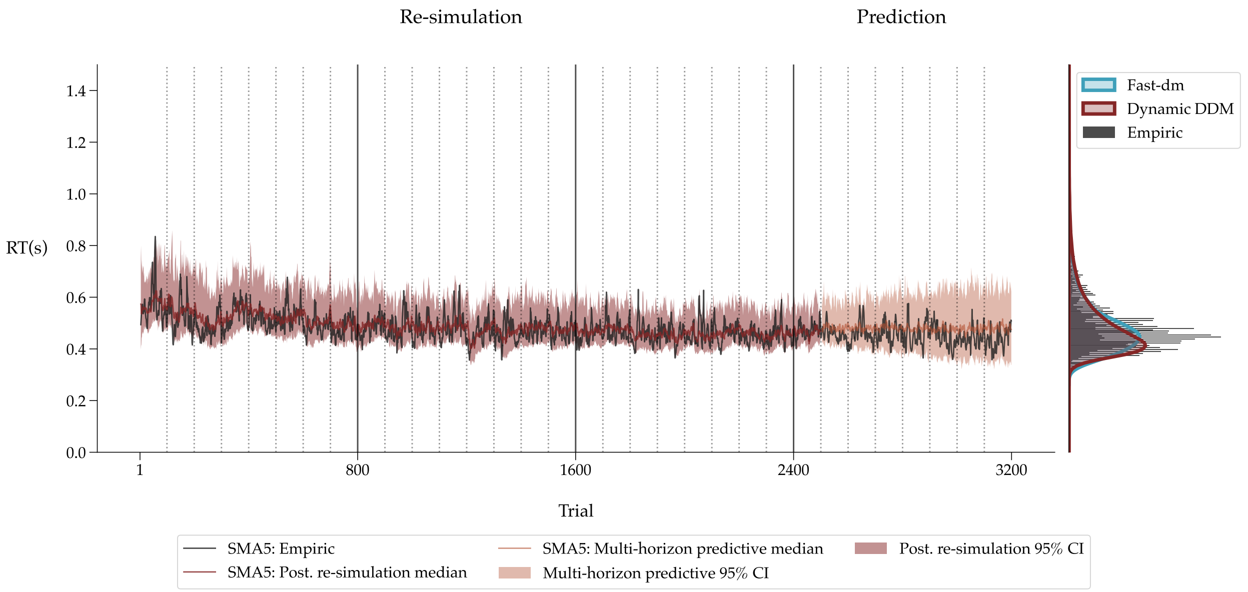

Figure A.28: **Left panel** The empirical RT time series of a single individual in black. From trial 1 to 2500, the median posterior re-simulation (aka *retrodictive check*) using the dynamic DDM is shown in red. The models' multi-horizon prediction is depicted for the remaining trials in orange. The shaded areas for the posterior re-simulation and prediction correspond to the 95% credibility interval. All the time series were smoothed via a simple moving average (SMA) with a period of 5. The dotted vertical lines indicate the end of an experimental block, and the solid vertical lines the end of an experimental session. **Right panel** The raw RT distribution is plotted as a histogram in black. The re-simulated RT distributions from the dynamic DDM and reference re-simulations from the static DDM using `fast-dm` are shown as kernel density estimates (KDEs) in red and blue, respectively.

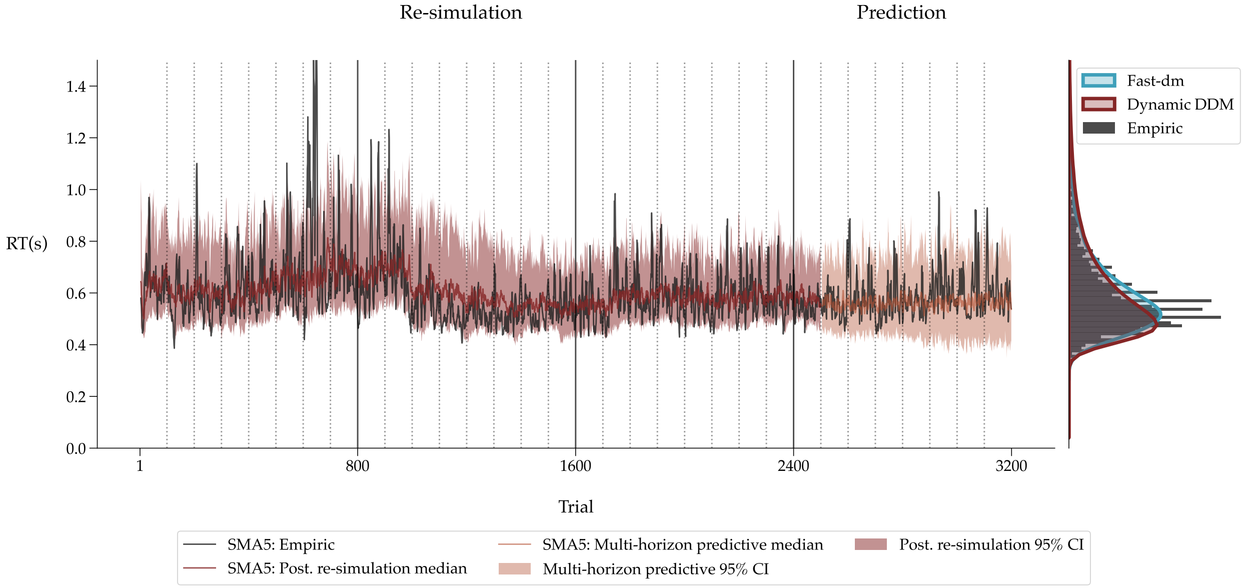

Figure A.29: **Left panel** The empirical RT time series of a single individual in black. From trial 1 to 2500, the median posterior re-simulation (aka *retrodictive check*) using the dynamic DDM is shown in red. The models' multi-horizon prediction is depicted for the remaining trials in orange. The shaded areas for the posterior re-simulation and prediction correspond to the 95% credibility interval. All the time series were smoothed via a simple moving average (SMA) with a period of 5. The dotted vertical lines indicate the end of an experimental block, and the solid vertical lines the end of an experimental session. **Right panel** The raw RT distribution is plotted as a histogram in black. The re-simulated RT distributions from the dynamic DDM and reference re-simulations from the static DDM using `fast-dm` are shown as kernel density estimates (KDEs) in red and blue, respectively.

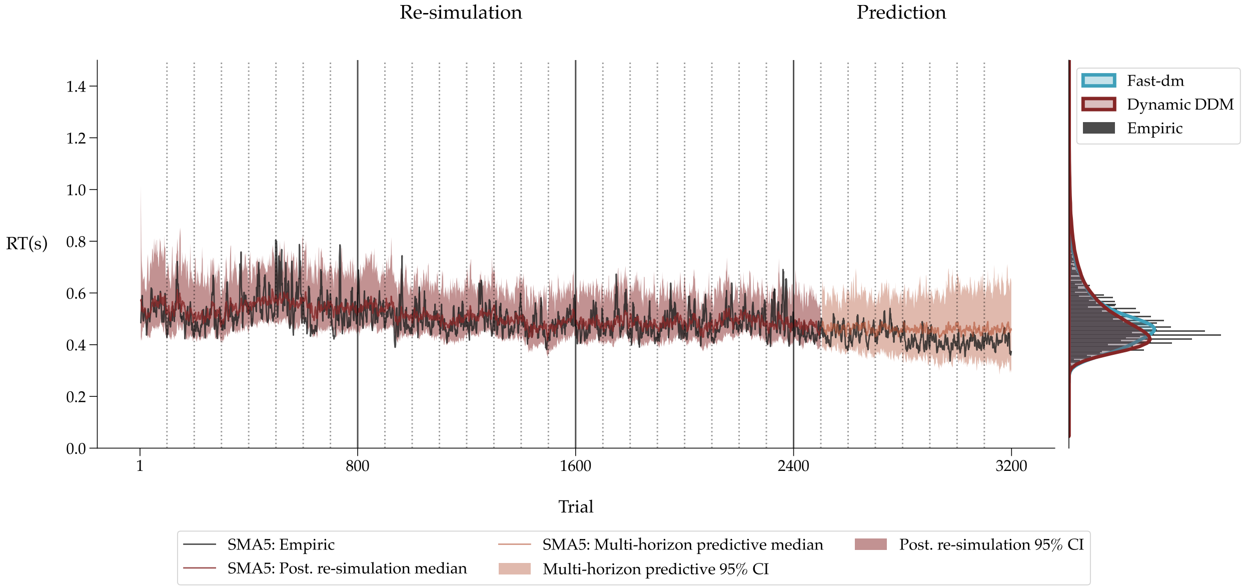

Figure A.30: **Left panel** The empirical RT time series of a single individual in black. From trial 1 to 2500, the median posterior re-simulation (aka *retrodictive check*) using the dynamic DDM is shown in red. The models' multi-horizon prediction is depicted for the remaining trials in orange. The shaded areas for the posterior re-simulation and prediction correspond to the 95% credibility interval. All the time series were smoothed via a simple moving average (SMA) with a period of 5. The dotted vertical lines indicate the end of an experimental block, and the solid vertical lines the end of an experimental session. **Right panel** The raw RT distribution is plotted as a histogram in black. The re-simulated RT distributions from the dynamic DDM and reference re-simulations from the static DDM using `fast-dm` are shown as kernel density estimates (KDEs) in red and blue, respectively.

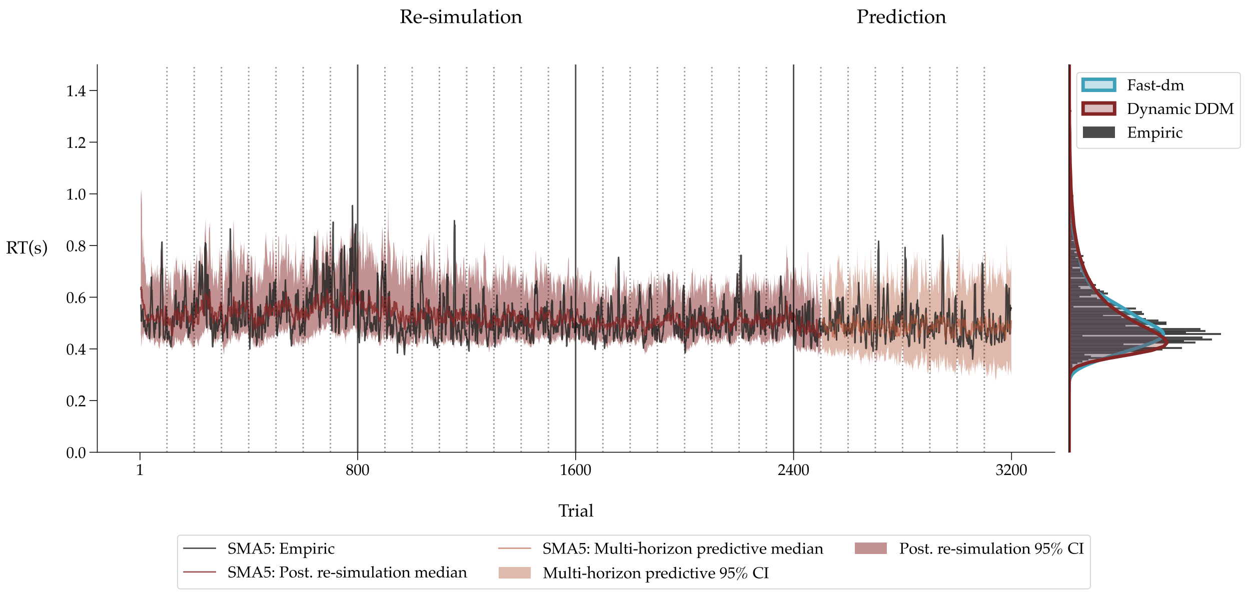

Figure A.31: **Left panel** The empirical RT time series of a single individual in black. From trial 1 to 2500, the median posterior re-simulation (aka *retrodictive check*) using the dynamic DDM is shown in red. The models' multi-horizon prediction is depicted for the remaining trials in orange. The shaded areas for the posterior re-simulation and prediction correspond to the 95% credibility interval. All the time series were smoothed via a simple moving average (SMA) with a period of 5. The dotted vertical lines indicate the end of an experimental block, and the solid vertical lines the end of an experimental session. **Right panel** The raw RT distribution is plotted as a histogram in black. The re-simulated RT distributions from the dynamic DDM and reference re-simulations from the static DDM using `fast-dm` are shown as kernel density estimates (KDEs) in red and blue, respectively.

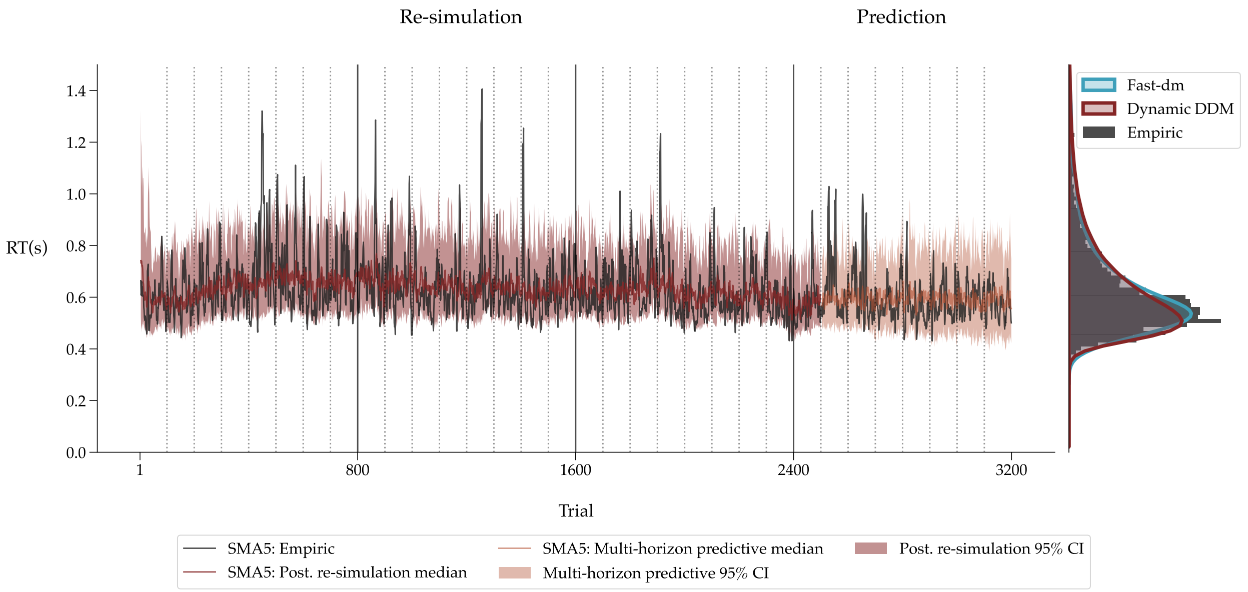

Figure A.32: **Left panel** The empirical RT time series of a single individual in black. From trial 1 to 2500, the median posterior re-simulation (aka *retrodictive check*) using the dynamic DDM is shown in red. The models' multi-horizon prediction is depicted for the remaining trials in orange. The shaded areas for the posterior re-simulation and prediction correspond to the 95% credibility interval. All the time series were smoothed via a simple moving average (SMA) with a period of 5. The dotted vertical lines indicate the end of an experimental block, and the solid vertical lines the end of an experimental session. **Right panel** The raw RT distribution is plotted as a histogram in black. The re-simulated RT distributions from the dynamic DDM and reference re-simulations from the static DDM using `fast-dm` are shown as kernel density estimates (KDEs) in red and blue, respectively.

Individual Parameter Dynamics

In the following, we show the inferred parameter dynamics of the remaining 10 participants not shown in the main text.

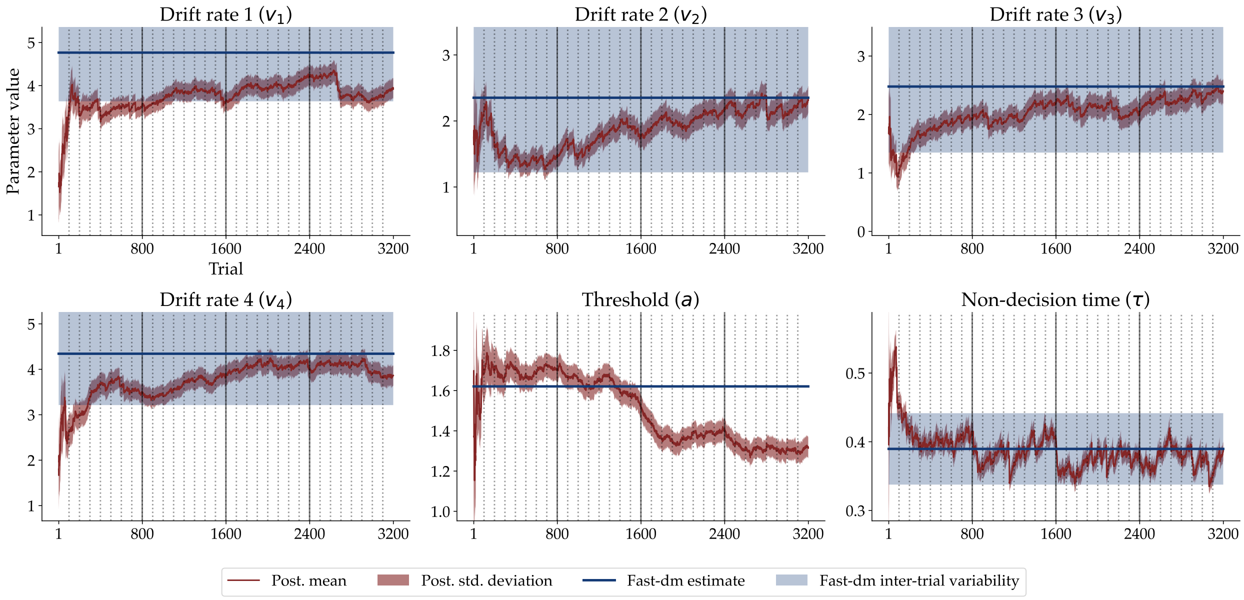

Figure A.33: The trial-wise posterior mean and ± 1 standard deviation for all six parameters, namely the four drift rates v_1 - v_4 (one for each experimental condition), the threshold a, and the non-decision time τ of an individual participant. The point estimates of the static DDM parameters and the corresponding inter-trial variabilities are shown in solid blue lines and blue shaded areas, respectively.

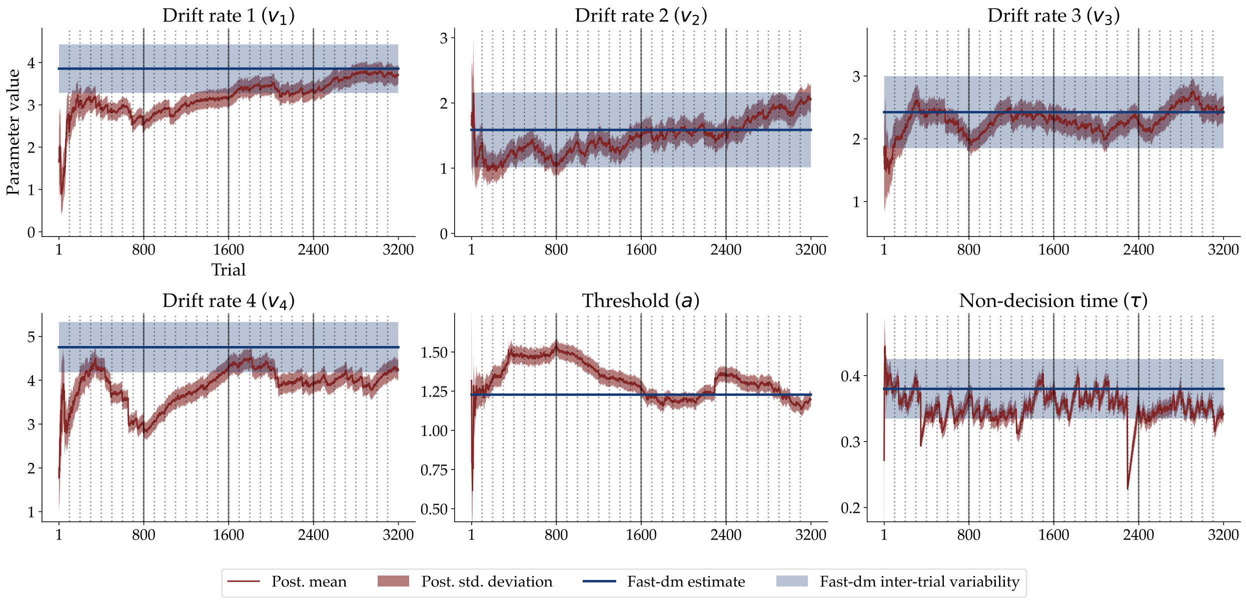

Figure A.34: The trial-wise posterior mean and ± 1 standard deviation for all six parameters, namely the four drift rates v_1 - v_4 (one for each experimental condition), the threshold a, and the non-decision time τ of an individual participant. The point estimates of the static DDM parameters and the corresponding inter-trial variabilities are shown in solid blue lines and blue shaded areas, respectively.

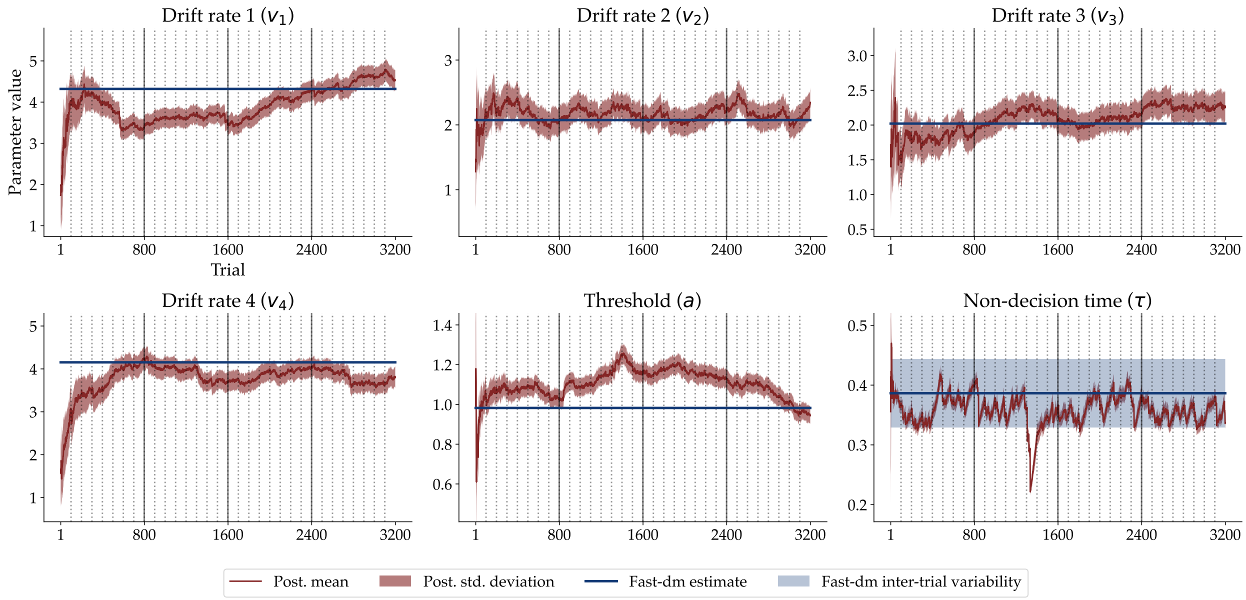

Figure A.35: The trial-wise posterior mean and ± 1 standard deviation for all six parameters, namely the four drift rates v_1 - v_4 (one for each experimental condition), the threshold a, and the non-decision time τ of an individual participant. The point estimates of the static DDM parameters and the corresponding inter-trial variabilities are shown in solid blue lines and blue shaded areas, respectively.

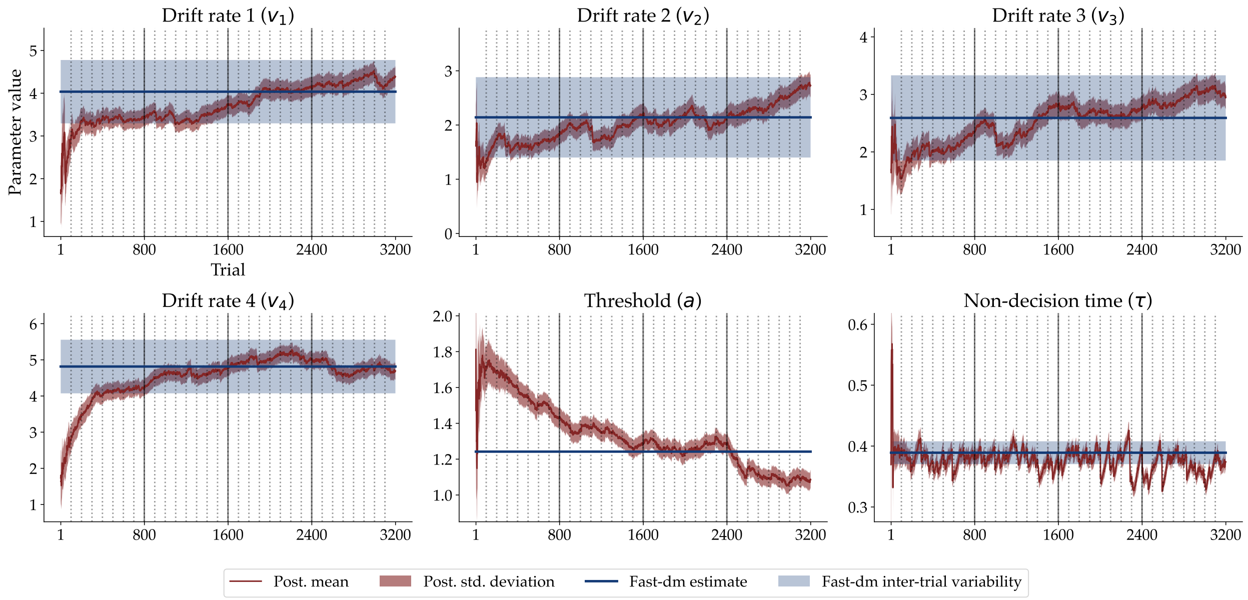

Figure A.36: The trial-wise posterior mean and ±1 standard deviation for all six parameters, namely the four drift rates v_1 - v_4 (one for each experimental condition), the threshold a, and the non-decision time τ of an individual participant. The point estimates of the static DDM parameters and the corresponding inter-trial variabilities are shown in solid blue lines and blue shaded areas, respectively.

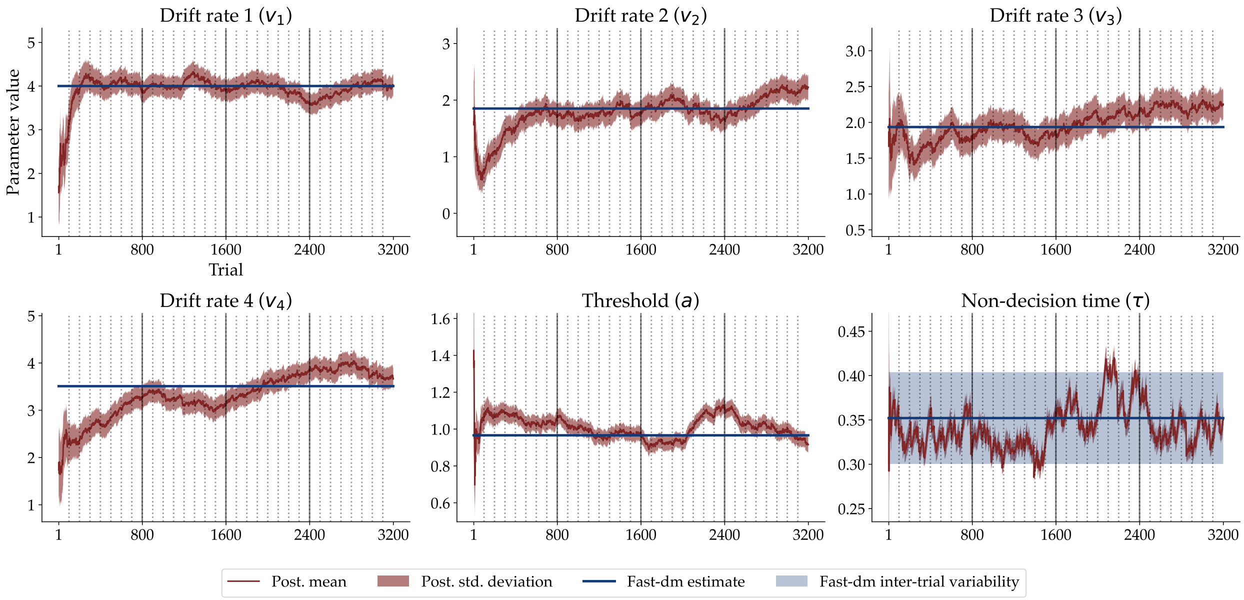

Figure A.37: The trial-wise posterior mean and ±1 standard deviation for all six parameters, namely the four drift rates v_1 - v_4 (one for each experimental condition), the threshold a, and the non-decision time τ of an individual participant. The point estimates of the static DDM parameters and the corresponding inter-trial variabilities are shown in solid blue lines and blue shaded areas, respectively.

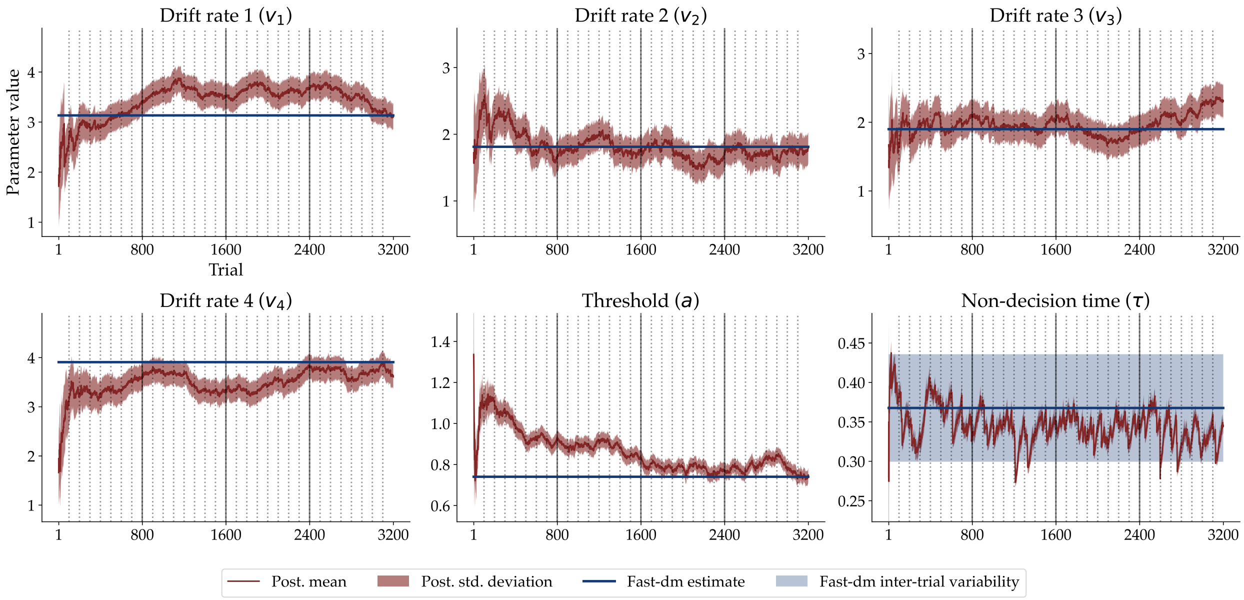

Figure A.38: The trial-wise posterior mean and ± 1 standard deviation for all six parameters, namely the four drift rates v_1 - v_4 (one for each experimental condition), the threshold a, and the non-decision time τ of an individual participant. The point estimates of the static DDM parameters and the corresponding inter-trial variabilities are shown in solid blue lines and blue shaded areas, respectively.

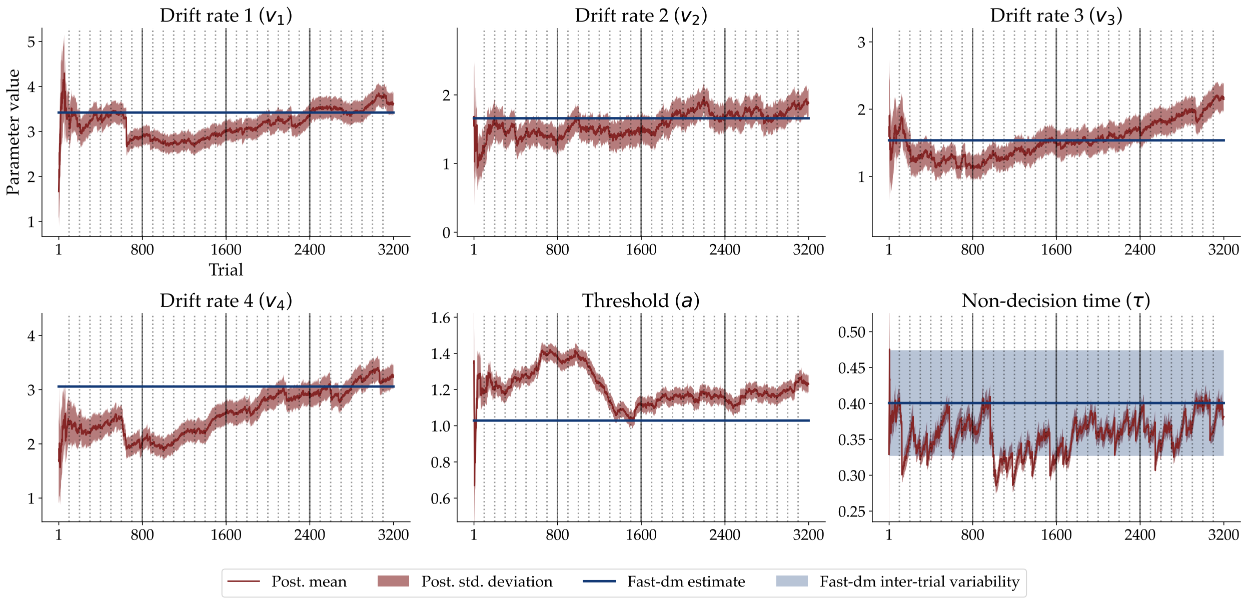

Figure A.39: The trial-wise posterior mean and ± 1 standard deviation for all six parameters, namely the four drift rates v_1 - v_4 (one for each experimental condition), the threshold a, and the non-decision time τ of an individual participant. The point estimates of the static DDM parameters and the corresponding inter-trial variabilities are shown in solid blue lines and blue shaded areas, respectively.

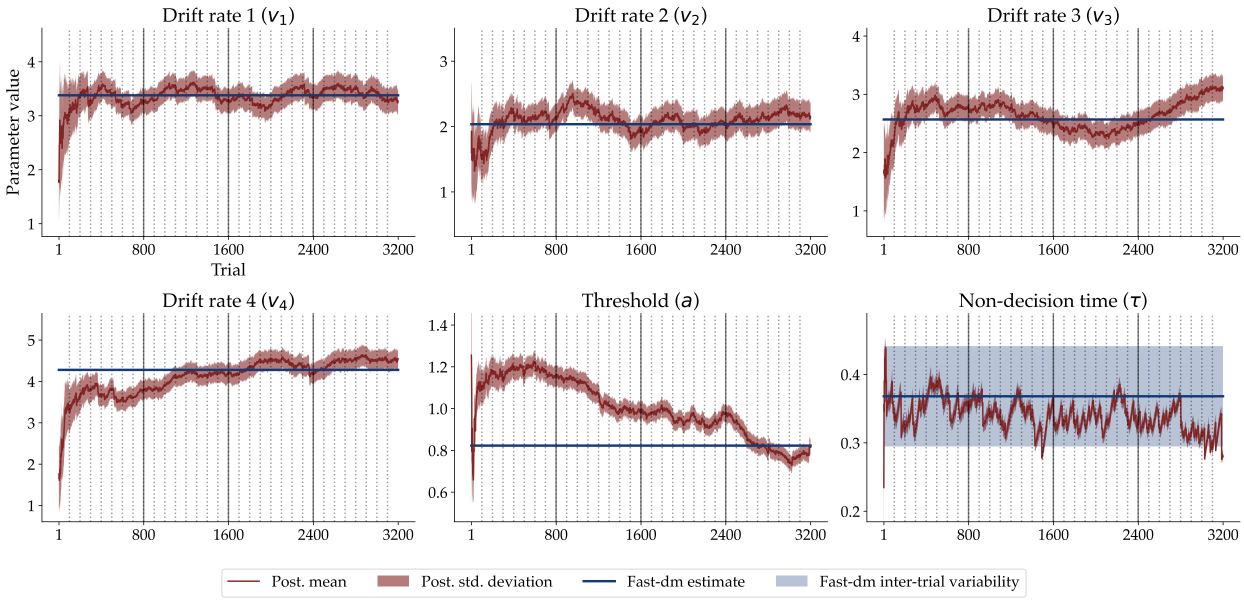

Figure A.40: The trial-wise posterior mean and ±1 standard deviation for all six parameters, namely the four drift rates v_1 - v_4 (one for each experimental condition), the threshold a, and the non-decision time τ of an individual participant. The point estimates of the static DDM parameters and the corresponding inter-trial variabilities are shown in solid blue lines and blue shaded areas, respectively.

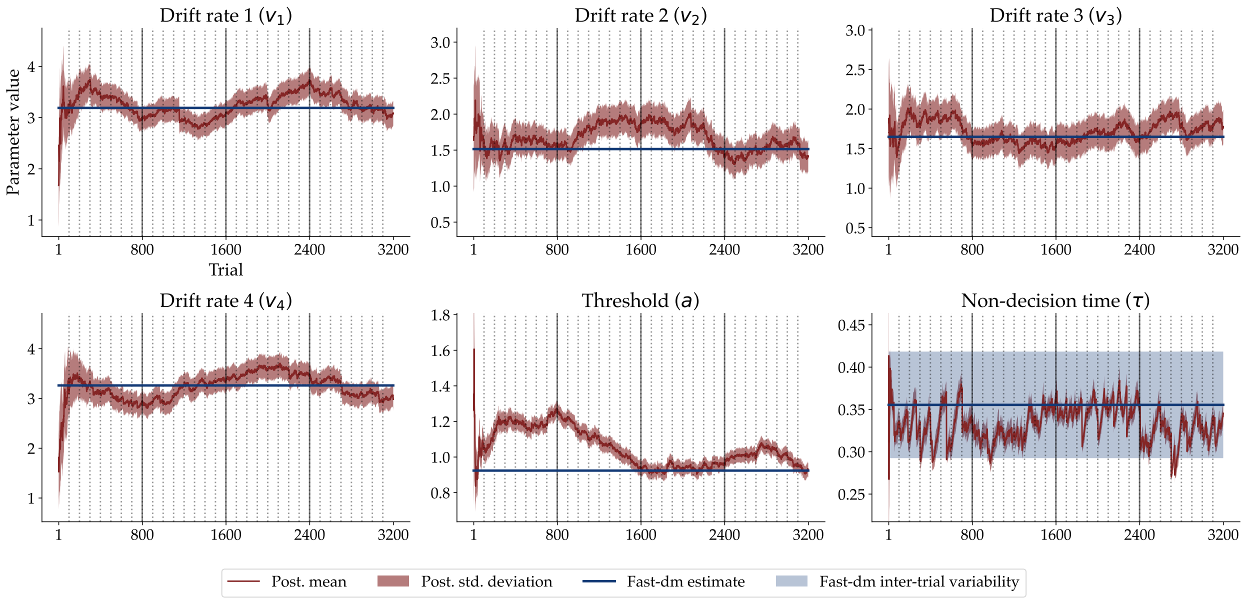

Figure A.41: The trial-wise posterior mean and ± 1 standard deviation for all six parameters, namely the four drift rates v_1 - v_4 (one for each experimental condition), the threshold a, and the non-decision time τ of an individual participant. The point estimates of the static DDM parameters and the corresponding inter-trial variabilities are shown in solid blue lines and blue shaded areas, respectively.

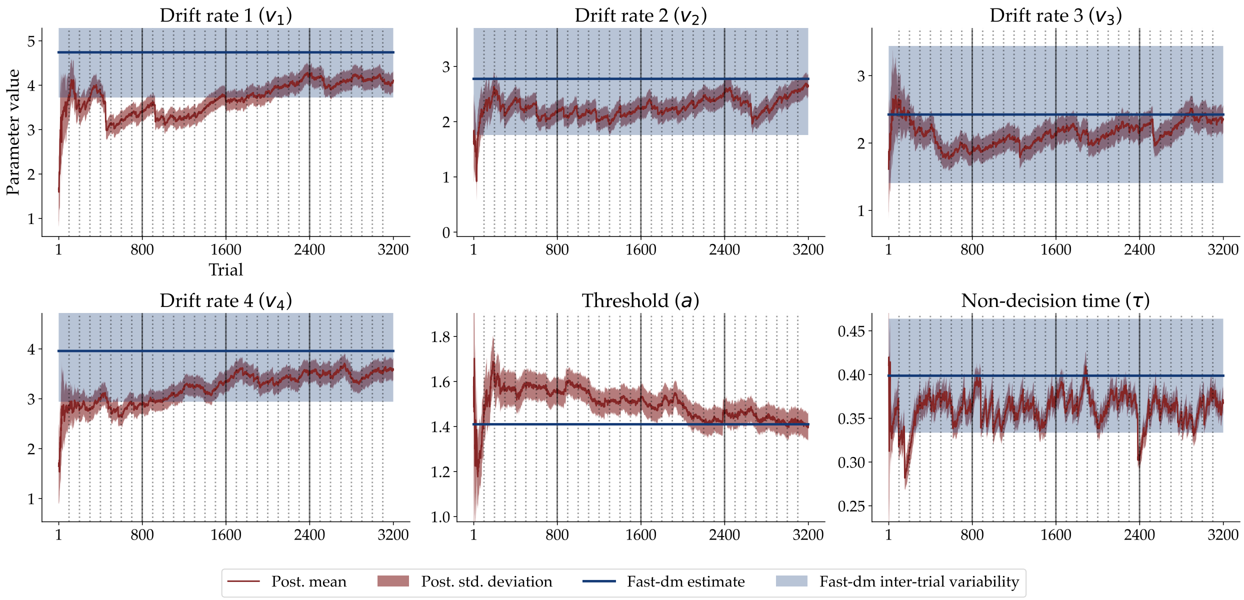

Figure A.42: The trial-wise posterior mean and ±1 standard deviation for all six parameters, namely the four drift rates v_1 - v_4 (one for each experimental condition), the threshold a, and the non-decision time τ of an individual participant. The point estimates of the static DDM parameters and the corresponding inter-trial variabilities are shown in solid blue lines and blue shaded areas, respectively.

Average Parameter Dynamics

Figure A.43 shows the parameter dynamic averaged across all participants.

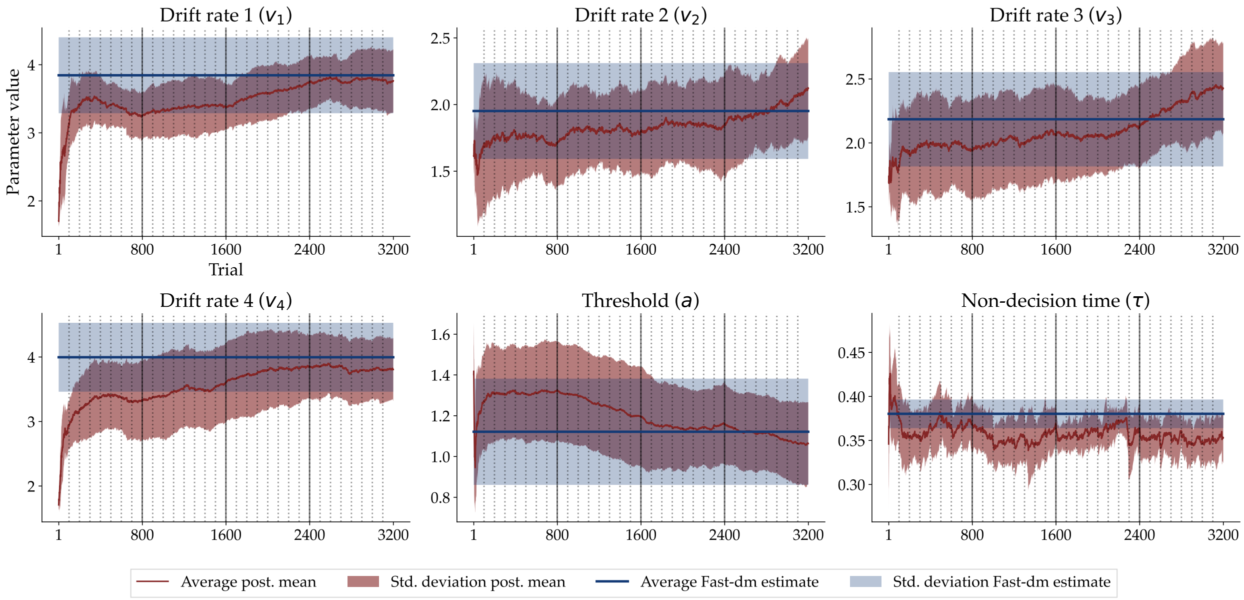

Figure A.43: The trial-wise posterior mean and ± 1 standard deviation for all six parameters, namely the four drift rates v_1 - v_4 (one for each experimental condition), the threshold a, and the non-decision time τ of averaged across all participant in solid red lines. The shaded red areas correspond to the ± 1 standard deviation of the posterior means of all individuals. The point estimates of the static DDM parameters averaged across all participants and the corresponding standard deviations are shown in solid blue lines and shaded blue areas, respectively.

Gaussian Random Walk Transition Model

We wanted to test if our neural estimation method can also estimate dynamic models with a simpler high-level transition model than a Gaussian process (GP). To this end, we fit a dynamic DDM with a Gaussian random walk as a transition model to the empirical data set described in the **Human data application** section:

$$\theta_t = T(\theta_{t-1}, \eta, z_t) = \theta_{t-1} + \eta \, z_t \quad \text{with} \quad z_t \sim \mathcal{N}(0, 1)$$

We use a Beta prior distribution parameterized with α and β for the standard deviations η_j of the Gaussian random walk transition model. The same prior distribution is used for all $j = 6$ low-level parameter transitions:

$$\eta_j \sim \text{Beta}(1, 25)$$

We trained the same neural network architecture as described in the main text for 50 epochs, 1000 batches per epoch, and a batch size of 8. The following figures show the results from simulation-based calibration (SBC), the model fit and inferred parameter dynamics for the same exemplar participant shown in the main text. Additionally, we depict the estimated parameter dynamics averaged across all individuals for comparison. These results are very similar to those obtained with the GP-DDM, which uses a Gaussian process as a transition model. However, the model with the Gaussian process transition model produces sharper predictions on unseen data. Note, that the dynamics implied by the random walk transition model are less sharper (i.e., contain more uncertainty) than those implied by the GP transition model.

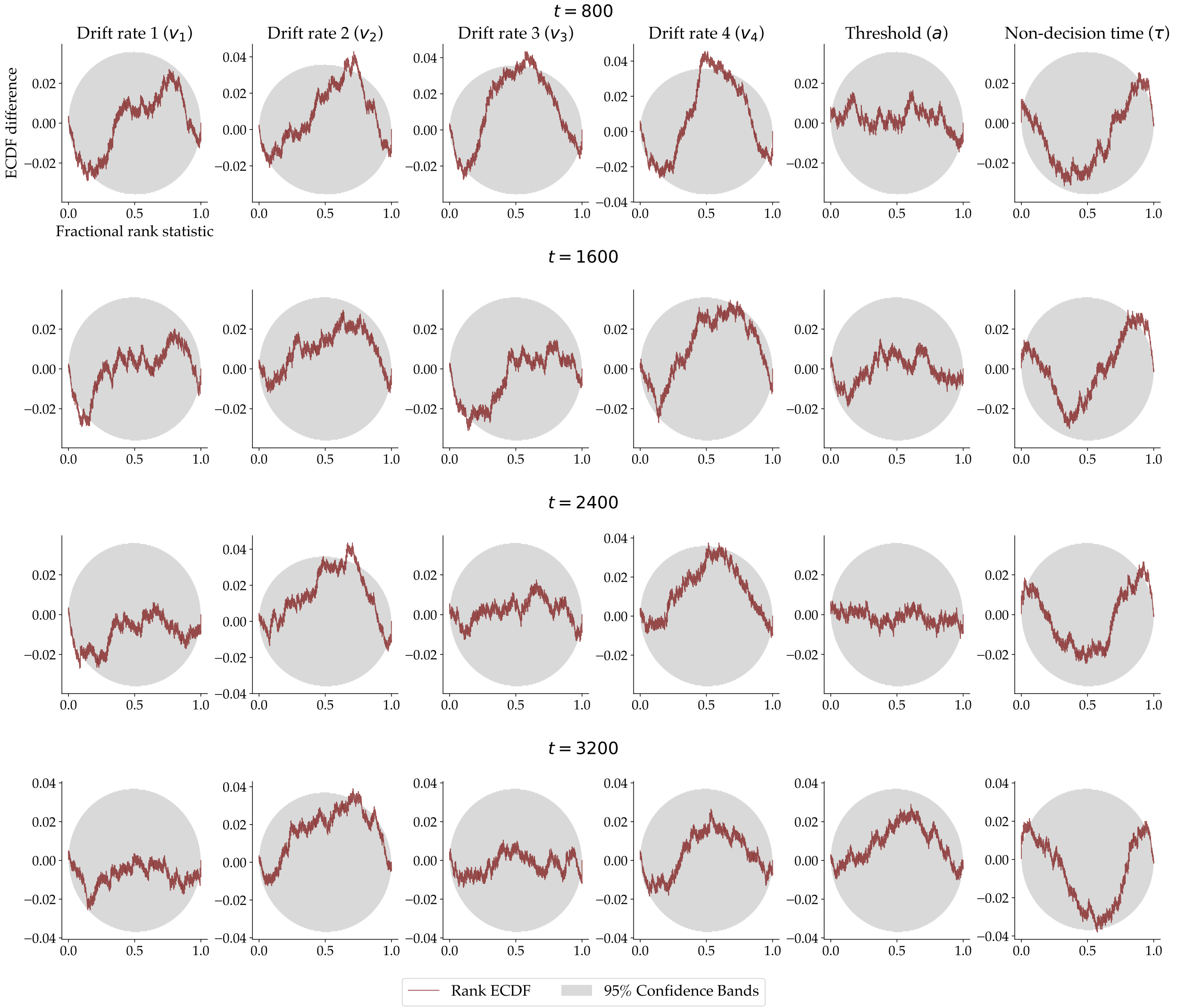

Figure A.44: **ECDF difference plot** 95% simultaneous confidence bands (gray) for the empirical cumulative distribution function (ECDF; red) for all 6 parameters at four selected time points (800, 1600, 2500, 3200) separately. We used the same settings as for the GP-DDM analysis.

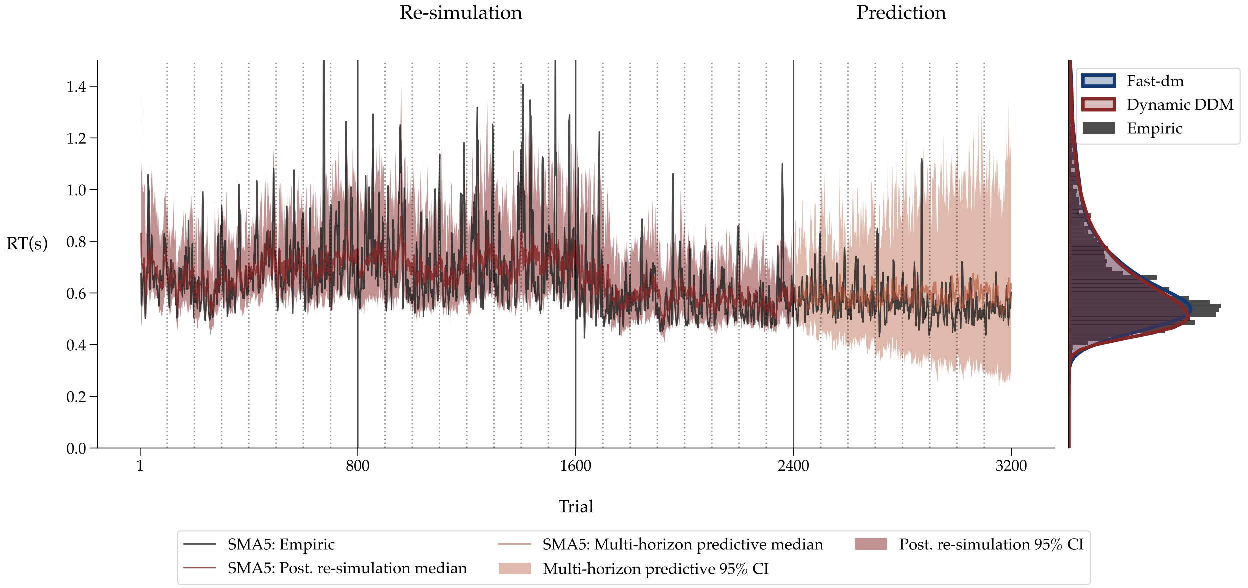

Figure A.45: **Left panel** The empirical RT time series of a single individual in black. From trial 1 to 2500, the median posterior re-simulation (aka *retrodictive check*) using the dynamic DDM is shown in red. The models' multi-horizon prediction is depicted for the remaining trials in orange. The shaded areas for the posterior re-simulation and prediction correspond to the 95% credibility interval. All the time series were smoothed via a simple moving average (SMA) with a period of 5. The dotted vertical lines indicate the end of an experimental block, and the solid vertical lines the end of an experimental session. **Right panel** The raw RT distribution is plotted as a histogram in black. The re-simulated RT distributions from the dynamic DDM and reference re-simulations from the static DDM using `Fast-dm` are shown as kernel density estimates (KDEs) in red and blue, respectively.

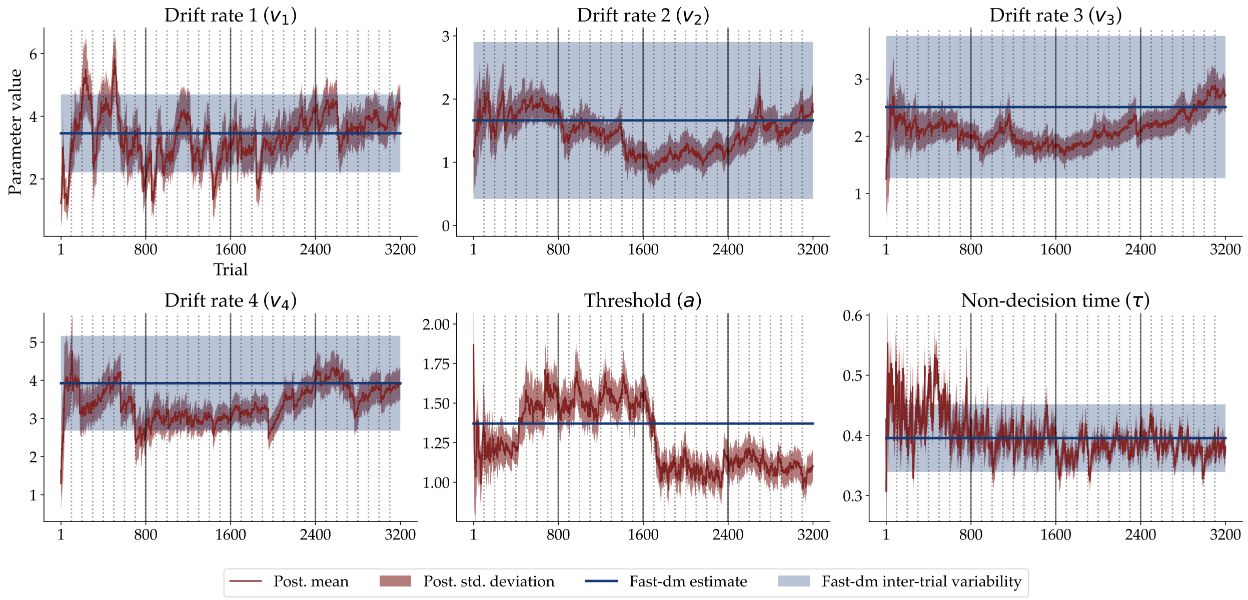

Figure A.46: The trial-wise posterior mean and ± 1 standard deviation for all six parameters, namely the four drift rates v_1 - v_4 (one for each experimental condition), the threshold a, and the non-decision time τ of an individual participant. The point estimates of the static DDM parameters and the corresponding inter-trial variabilities are shown in solid blue lines and blue shaded areas, respectively.

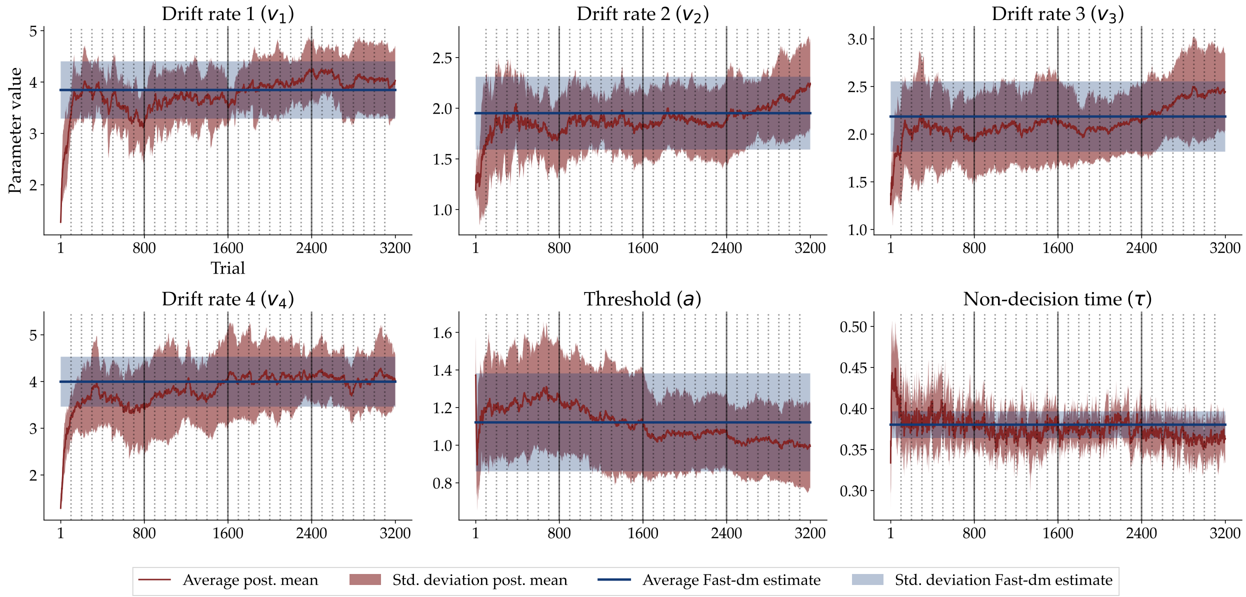

Figure A.47: The trial-wise posterior mean and ± 1 standard deviation for all six parameters, namely the four drift rates v_1 - v_4 (one for each experimental condition), the threshold a, and the non-decision time τ of averaged across all participant in solid red lines. The shaded red areas correspond to the ± 1 standard deviation of the posterior means of all individuals. The point estimates of the static DDM parameters averaged across all participants and the corresponding standard deviations are shown in solid blue lines and shaded blue areas, respectively.
